# Prostate-specific membrane antigen (PSMA)-targeted photodynamic therapy enhances the delivery of PSMA-targeted magnetic nanoparticles to PSMA-expressing prostate tumors

**DOI:** 10.7150/ntno.52361

**Published:** 2021-01-19

**Authors:** Ethel J. Ngen, Ying Chen, Babak Behnam Azad, Srikanth Boinapally, Desmond Jacob, Ala Lisok, Chentian Shen, Mir S. Hossain, Jiefu Jin, Zaver M. Bhujwalla, Martin G. Pomper, Sangeeta R. Banerjee

**Affiliations:** 1 The Russell H. Morgan Department of Radiology and Radiological Science, Johns Hopkins University School of Medicine, Baltimore, MD 21287, USA.; 2 The Sidney Kimmel Comprehensive Cancer Center, Johns Hopkins University School of Medicine, Baltimore, MD 21231, USA.; 3 The F. M. Kirby Research Center for Functional Brain Imaging, Kennedy Krieger Research Institute, Baltimore, Maryland 21205, USA.

**Keywords:** prostate cancer, magnetic nanoparticle delivery, photodynamic therapy (PDT), enhanced permeability and retention (EPR) effect, magnetic resonance imaging (MRI)

## Abstract

Enhanced vascular permeability in tumors plays an essential role in nanoparticle delivery. Prostate-specific membrane antigen (PSMA) is overexpressed on the epithelium of aggressive prostate cancers (PCs). Here, we evaluated the feasibility of increasing the delivery of PSMA-targeted magnetic nanoparticles (MNPs) to tumors by enhancing vascular permeability in PSMA(+) PC tumors with PSMA-targeted photodynamic therapy (PDT).

**Method:** PSMA(+) PC3 PIP tumor-bearing mice were given a low-molecular-weight PSMA-targeted photosensitizer and treated with fluorescence image-guided PDT, 4 h after. The mice were then given a PSMA-targeted MNP immediately after PDT and monitored with fluorescence imaging and T_2_-weighted magnetic resonance imaging (T_2_-W MRI) 18 h, 42 h, and 66 h after MNP administration. Untreated PSMA(+) PC3 PIP tumor-bearing mice were used as negative controls.

**Results:** An 8-fold increase in the delivery of the PSMA-targeted MNPs was detected using T_2_-W MRI in the pretreated tumors 42 h after PDT, compared to untreated tumors. Additionally, T_2_-W MRIs revealed enhanced peripheral intra-tumoral delivery of the PSMA-targeted MNPs. That finding is in keeping with two-photon microscopy, which revealed higher vascular densities at the tumor periphery.

**Conclusion:** These results suggest that PSMA-targeted PDT enhances the delivery of PSMA-targeted MNPs to PSMA(+) tumors by enhancing the vascular permeability of the tumors.

## Introduction

Prostate cancer (PC) is the most prevalent non-cutaneous cancer among men in the United States 1. Standard therapies such as radical prostatectomy and radiation therapy often result in substantial morbidity, which negatively impacts quality of life 2, 3. Focal therapies are currently being explored for the treatment of patients with intermediate and high-risk PC, localized within the prostate capsule 4, 5.

Magnetic nanoparticle (MNP)-induced hyperthermia is a focal therapy currently being explored to treat localized, intermediate, and high-risk PC 6, 7. In addition to hyperthermia, MNPs can serve as magnetic resonance imaging (MRI) contrast agents and magnetic particle imaging (MPI) probes for image guidance during therapy, as well as drug and gene delivery vehicles 8-12. Current preclinical and clinical practices to deliver high doses of MNPs to tumors for hyperthermia therapy involve directly injecting MNPs into tumors 6, 13. Unfortunately, direct tumor injection is an invasive procedure that can cause treatment-related morbidity and subsequently negatively impact patient quality of life after treatment 10. In addition, given the multi-focal nature of PC lesions, direct injections could miss remote or nearby foci. Consequently, there is a need for the development of MNP delivery strategies capable of increasing tumor-specific accumulation of MNPs, after intravenous administration.

Although several receptor-targeted nanoparticles have been developed, their effective delivery to tumors is still a major challenge, especially for nanoparticles ≥ 100 nm in diameter 14, 15. Interestingly, recent studies suggest that MNPs ranging between 100 - 200 nm in diameter, with high iron content, and capable of specifically targeting tumors, are highly desirable for MRI-guided hyperthermia 16-20. Enhanced vascular permeability in tumors has been shown to play an essential role in the delivery of nanoparticles of this diameter range to tumors through the enhanced permeability and retention (EPR) effect 21, 22. Additionally, the poor clinical performance of some receptor-targeted nanoparticles has been partially attributed to variable vascular permeability within tumors 14, 15. Consequently, targeted tumor-priming strategies designed specifically to enhance tumor vascular permeability and subsequently increase the delivery of nanoparticles to tumors are currently being explored 22-26. Anti-angiogenic therapies have also been reported to have tumor vessel normalizing effects, which increase the delivery of therapeutic agents to tumors and subsequently improve therapeutic outcomes 26-31. Receptor-targeted photoimmunotherapy (PIT) is an example of a tumor-priming strategy that has been used preclinically to enhance tumor vascular permeability in different tumor phenotypes by specifically destroying tumor cells in the perivascular space while leaving tumor vessels intact after therapy 25, 32-35. This targeted destruction of tumor cells in the perivascular space enables an increase in tumor blood flow and nanoparticle perfusion of the tumor blood vessels and subsequently increases nanoparticle delivery to tumors 25, 32-35. However, one of the limitations of PIT is that it involves the use of antibodies with unfavorable pharmacokinetic profiles, slow clearance rates, and potential immunotoxicity 36. Photodynamic therapy (PDT) is an alternative light-activated therapy which, although similar to PIT, does not require the use of antibody-conjugated photosensitizers 35, 37-42. Furthermore, PDT using an untargeted lipophilic photosensitizer was previously shown to increase nanoparticle delivery to tumors 42. Vascular-targeted PDT is a focal therapy that is also currently being explored for the treatment of localized PC 43, 44. Consequently, we postulated that PDT using a receptor-targeted low-molecular-weight ligand conjugated to a photosensitizer could be used specifically to enhance tumor vascular permeability and subsequently the delivery of receptor-targeted MNPs to prostate tumors.

Prostate-specific membrane antigen (PSMA) is a type II integral membrane glycoprotein that is overexpressed on the neovasculature of solid tumors and on the epithelium of aggressive PCs 45, 46. Based on our studies using a PSMA-based antibody and low-molecular-weight PSMA-based compounds, we previously demonstrated that the low-molecular-weight PSMA-targeted compounds had more favorable pharmacokinetics than the antibody and provided rapid and high tumor uptake and clearance from non-target tissues 47-49. Accordingly, we hypothesized that our low-molecular-weight PSMA-targeted photosensitizer would follow a similar mechanism to PIT, but with superior pharmacokinetics, since both our agent and the PIT agents target the tumor epithelium 50.

**Figure 1:**
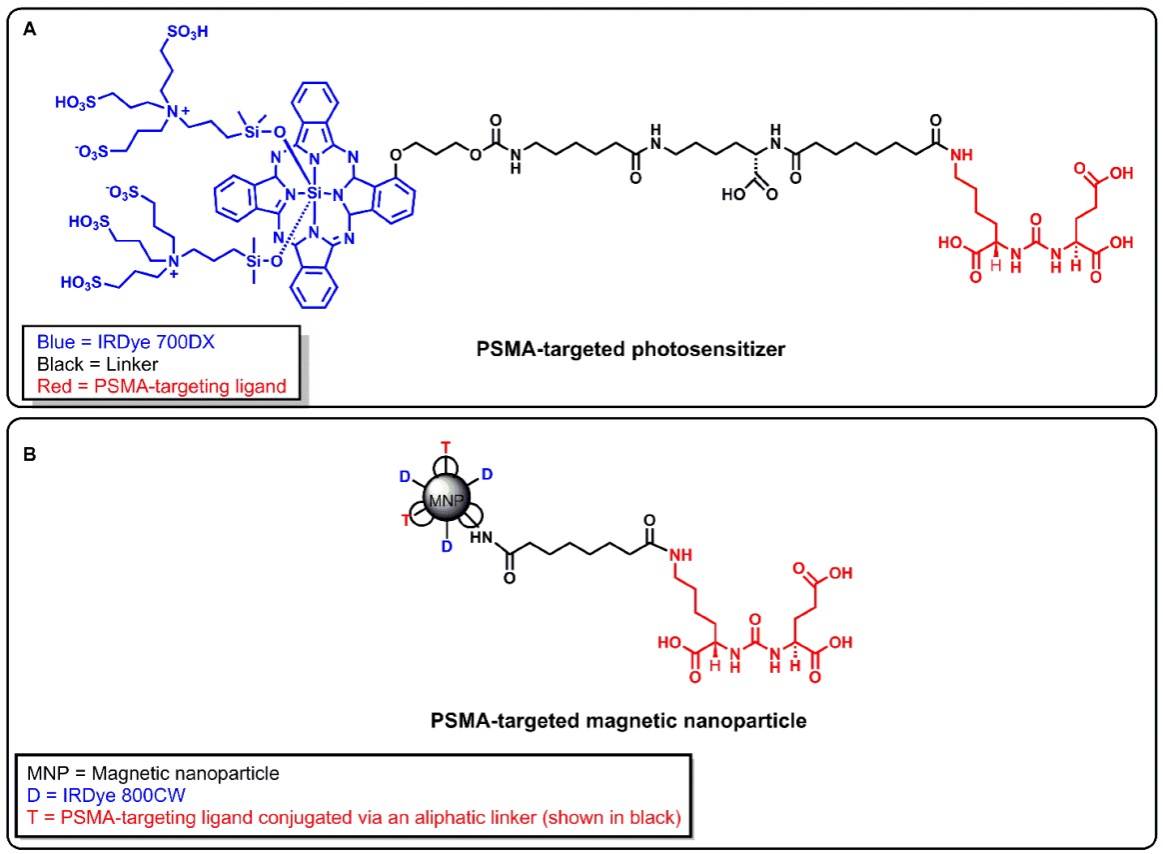
** A)** Structure of a PSMA-targeted photosensitizer YC-9. **B)** Structure of a PSMA-targeted magnetic nanoparticle (MNP). A low-molecular-weight PSMA-targeting ligand Glu-Lys-urea-suberate was used in both agents to target PSMA.

Recently, we developed a PSMA-targeted photosensitizer (YC-9) and demonstrated that it could effectively control the growth of PSMA-expressing tumors, long-term, after four 10 nmol doses of YC-9 and PDT at 48 h intervals 50. YC-9 was synthesized by conjugating a low-molecular-weight PSMA ligand, Glu-Lys-urea-suberate((((*S*)-1-carboxy-5-(7-carboxyheptanamido)pentyl)carbamoyl)-L-glutamic acid), to a near-infrared photosensitizer (IRDye^®^ 700DX), via a flexible linker 50. We also recently developed a PSMA-targeted MNP and demonstrated the feasibility of preferentially targeting PSMA(+) tumors compared to PSMA(-) tumors at low and high MNP concentrations in preclinical PC models with high vascular permeability 51, 52. To test our hypothesis, here we evaluated the feasibility of using YC-9 (Figure 1A) for PSMA-targeted PDT to specifically enhance tumor vascular permeability and the delivery of our previously developed PSMA-targeted MNP (Figure 1B) to PSMA-expressing tumors with low vascular permeability.

## Methods

### Materials

Reagents and solvents were purchased from either Sigma-Aldrich (Milwaukee, WI) or Fisher Scientific (Pittsburgh, PA) unless otherwise stated. LI-COR IRDye^®^ 700DX NHS ester and LI-COR IRDye^®^ 800CW NHS ester were purchased from LI-COR Biosciences (Lincoln, NE). Starch-coated, amine-surface-modified bionized nanoferrite particles (80 nm in diameter) were obtained from Micromod Partikeltechnologie GmbH (Rostock, Germany) and were used as the magnetic nanoparticle (MNP) core. All MNPs were PEGylated using *N*-hydroxylsuccinimide (NHS) bifunctional polyethylene glycol crosslinkers (NHS-PEG1000-NHS) obtained from Creative PEG Works (Chapel Hill, NC). A PSMA-targeted photosensitizer (YC-9), previously developed by our group, was synthesized and characterized as previously reported and used in this study 50. A PSMA-targeted MNP (hydrodynamic diameter = 147 ± 8 nm and ζ-potential = -10.9 ± 0.3 mV), also previously developed by our group, was synthesized and characterized as previously reported, and used in this study 51, 52. The characterization of the synthesized MNPs previously reported is also included in the Supplementary Material (Methods S1, S2, Table S1, and Figure S1) 51, 52.

### Instrumentation

All PDT studies were performed using a 690 ± 20 nm light-emitting diode (LED) source (L690-66-60), obtained from Marubeni America Corporation (Santa Clara, CA). All MRI scans were performed on a Bruker Biospec 11.7T horizontal bore scanner, equipped with a quadrature proton resonator radiofrequency coil (RF RES 500 ^1^H 075/040 QSN TR), for 500 MHz MR systems (Billerica, MA). All animal fluorescence imaging was performed on a LI-COR Pearl^®^ Trilogy small animal imaging system (Lincoln, NE). Bright-field optical microscopy images were acquired using an Aperio ScanScope^®^ AT system (Vista, CA). Multi-photon microscopy images were acquired on an Olympus Laser Scanning FV1000 microscope equipped with a 25xw/1.05XLPLN MP lens and obtained from Olympus Corporation (Center Valley, PA).

### Cell lines

Androgen-independent PC3 human prostate tumor xenograft-derived cell sublines were used in all studies. The sublines were generously provided by Dr. Warren Heston (Cleveland Clinic). These cells were genetically engineered either to express high levels of PSMA [PSMA(+) PC3 PIP cells] or not to express PSMA [PSMA(-) PC3 flu cells] 53, 54. Both PSMA(+) PC3 PIP and PSMA(-) PC3 flu prostate cancer cell lines were cultured in RPMI 1640 medium (Corning Cellgro, Manassas, VA) containing 10% fetal bovine serum (FBS) (Invitrogen, Carlsbad, CA) and 1% penicillin-streptomycin (Corning Cellgro, Manassas, VA), as previously described 54, 55. All cell cultures were maintained in 5% carbon dioxide (CO_2_) at 37°C in a humidified incubator.

### Mouse experimental design

All animal experiments were carried out according to the Johns Hopkins University Animal Care and Use Committee guidelines. Five-to-six week-old, male non-obese diabetic severe-combined immunodeficient gamma (NSG) mice, obtained from the Johns Hopkins University Animal Resources Core, were used for all studies. Mice were subcutaneously inoculated with 3 × 10^6^ PSMA(+) PC3 PIP (right upper flanks) and/or 1 × 10^6^ PSMA(-) PC3 flu cells (left upper flanks). Tumor growth was monitored using calipers until the xenografts reached volumes of ~50 mm^3^. Tumor volumes were calculated using the modified ellipsoidal formula: (length × width^2^)/2. Hair removal cream (Reckitt Benckiser Inc., Parsippany, NJ) was applied to the fur surrounding the tumor region, and the fur was removed prior to all PDT studies.

**Scheme 1:**
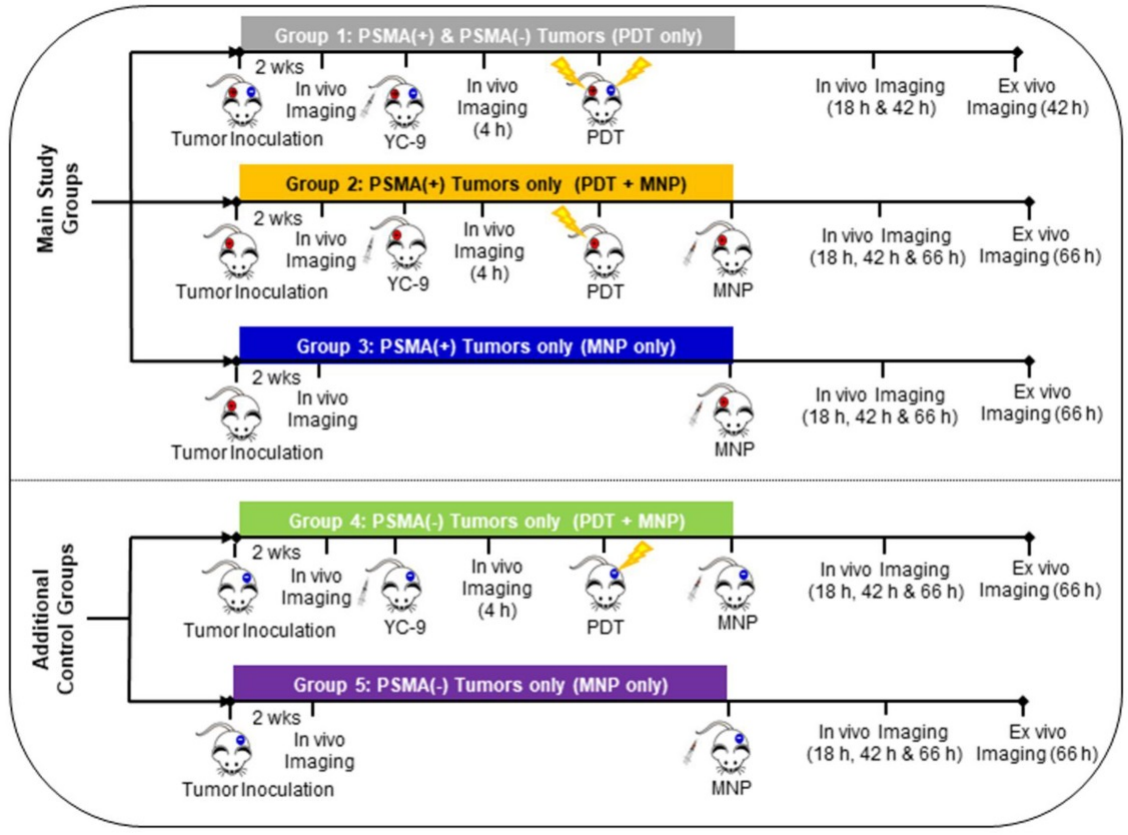
Experimental design of the different mouse groups.

*In vivo* experiments were then performed as demonstrated in Scheme 1. Five groups of mice were used in this study. Group 1: Mice bearing both PSMA(+) PC3 PIP and PSMA(-) PC3 flu tumors and treated with PDT only and then imaged sequentially. Group 2: Mice bearing only PSMA(+) PC3 PIP tumors and pretreated with PDT before MNP administration, then imaged sequentially. Group 3: Mice bearing only PSMA(+) PC3 PIP tumors and only administered MNPs, then imaged sequentially. Group 4: Mice bearing only PSMA(-) PC3 flu tumors and pretreated with PDT before MNP administration, then imaged sequentially. Group 5: Mice bearing only PSMA(-) PC3 flu tumors and only administered MNPs, then imaged sequentially. Group 4 and 5 mice, bearing only PSMA(-) PC3 flu tumors, were studied as additional control groups.

Mice bearing both PSMA(+) PC3 PIP and PSMA(-) PC3 flu tumors (Group 1) were used only as controls to ensure that PDT had similar effects on the T_2_W MRI signal changes in both tumor phenotypes and that the PDT effects did not interfere with the detection of MNPs on T_2_W MRIs in both tumor phenotypes. To evaluate the effect of PDT on enhancing MNP delivery, mice bearing either PSMA(+) PC3 PIP or PSMA(-) PC3 flu tumors were used, and direct comparisons were made between the PDT treated group versus the untreated group for each tumor phenotype. This was done because PSMA(-) PC3 flu tumors have a much faster growth rate than PSMA(+) PC3 PIP. Even when fewer numbers of PSMA(-) PC3 flu cells are inoculated at a much later time-point in the same mouse, PSMA(+) PC3 PIP and PSMA(-) PC3 flu tumors do not always develop to the same volume at the desired experimental start time.

In addition, we had previously demonstrated that there was a direct correlation between the tumor volume and the tumor EPR effect in both PSMA(+) PC3 PIP and PSMA(-) PC3 flu tumors. In large PSMA(+) PC3 PIP and PSMA(-) PC3 flu tumors, we noticed higher EPR effects, and this resulted in an increase in MNP delivery to both tumors 52 While in smaller PSMA(+) PC3 PIP and PSMA(-) PC3 flu tumors, the EPR effect, and MNP delivery was reduced in both tumors 52. Thus, given the difference in tumor growth rates and the correlation between tumor volume and the EPR effect, mice with single small tumors were used to minimize variations in the tumor volumes and the EPR effect between the different tumor phenotypes.

To evaluate the non-specific contributions of PDT to enhance MNP delivery, PDT pretreated PSMA(-) PC3 flu Group 4 mice were compared to untreated PSMA(-) PC3 flu Group 5 mice. The contribution of PSMA-targeted PDT to enhance MNP delivery was indirectly evaluated by comparing the difference between the PSMA(+) PC3 PIP groups and the PSMA(-) PC3 flu groups [(Group 2 versus Group 3) compared to (Group 4 versus Group 5)].

Only data from the main experimental groups (Groups 2 and 3), and one control group (Group 1) is included in the main manuscript due to space constraints. Data from the additional control groups (Groups 4 and 5) are included in the Supplementary Material. The description of all previously reported experimental methods used in this study, are also included in the Supplementary Material.

### *In vivo* fluorescence imaging

Fluorescence image guidance was used to validate the time-point after the administration of the PSMA-targeted photosensitizer (YC-9) at which to irradiate the mice with near-infrared (NIR) light for PDT. The right time for PDT irradiation was determined from our previous studies using YC-9 and our other low molecular weight (LMW) PSMA-targeting agents. This showed that optimum PSMA targeting is achieved four to six hours after the administration of these LMW PSMA-targeted agents 50, 55. That window became the time for PDT irradiation. YC-9 was imaged using the LI-COR IR Dye^®^ 700DX, contained in its structure (Figure 1A), at a fixed excitation wavelength (λ_ex_) of 685 nm and emission wavelength (λ_em_) of 700 nm.

Fluorescence image guidance was also used to detect nanoparticle accumulation in the tumors 18 h, 42 h, and 66 h after PDT and MNP administration. These time-points were determined from our previous studies with the same PSMA-targeted MNP. We demonstrated that optimum MNP accumulation in tumors was detected around 24 h to 48 h after the intravenous administration of the PSMA-targeted MNP 51, 52. PDT was performed 4 h after the administration of YC-9, so the MNP imaging Time-points were adjusted to 18 h, 42 h, and 66 h after PDT and MNP administration. This imaging time-point adjustment also took into account two hours of PDT irradiation for mice with both PSMA(+) and PSMA(-) tumor phenotypes. The PSMA-targeted MNP was imaged using the LI-COR IR Dye^®^ 800CW contained in its structure (Figure 1B), at a fixed λ_ex_ = 785 nm and λ_em_ = 800 nm.

All fluorescence imaging was performed on mice under anesthesia, using 2% isoflurane in an oxygen and air mixture. Mice were imaged using a LI-COR Pearl^®^ Trilogy small animal imaging system. Images were acquired at a resolution of 170 μm. The fluorescence images were then quantified using the LI-COR Pearl^®^ Trilogy small animal imaging software, version 2.0.

### *In vivo* photodynamic therapy (PDT)

Mice in the PDT pretreatment groups were irradiated with NIR light from a 690 ± 20 nm light-emitting diode (LED) source, 4 h after the administration of 3.3 nmol of the PSMA-targeted photosensitizer, YC-9. This dose was determined from our previous study with YC-9, where four cycles of 10 nmol of YC-9 and PDT at 48 h intervals were needed to achieve complete tumor eradication 50. Although a single PDT treatment cycle after a YC-9 dose of 10 nmol caused a reduction in the tumor volume in that study, it did not completely eradicate the tumors 50. Accordingly, to investigate the feasibility of using this PDT method to enhance tumor vascular permeability and nanoparticle delivery, we decided to use a lower YC-9 dose that did not cause a reduction in the tumor volume in our previous study 50.

Briefly, 4 h after YC-9 administration, each mouse was covered with aluminum foil, except at the tumor site, to prevent non-specific activation of the photosensitizer in other parts of the body. The tumors were then irradiated at a total fluence of 200 J/cm^2^. Mice were then imaged 18 h, 42 h, and 66 h after PDT (24 h, 48 h, and 72 h after YC-9 administration, respectively).

### *In vivo* MRI

MRI was used to detect *in vivo* MNP delivery to tumors at a higher spatial resolution than fluorescence imaging. The high spatial resolution of MRI was also used to elucidate the intra-tumoral distribution of the delivered MNPs, at the different time-points after MNP delivery. *In vivo,* MRI experiments were performed under anesthesia using 2% isoflurane in an air and oxygen mixture. T_2_-weighted (T_2_-W) MRIs were acquired before, 18 h; 42 h, and 66 h after the bolus intravenous administration of 50 mg/kg of the PSMA-targeted MNPs (30 mg/kg of Fe). T_2_-W MRIs were acquired using a spin echo, rapid acquisition with refocused echoes (RARE) pulse sequence. Parameters: echo time (TE) = 10 ms; effective echo time (TE_Eff_) = 30 ms; RARE factor = 8; repetition time (TR) = 4,000 ms; number of averages (NA) = 2; field of view (FOV) = 25 × 25 mm; matrix size (MS) = 128 × 128 pixels; and slice thickness = 0.5 mm. All image analyses were performed using NIH ImageJ software. The T_2_-W MRI signal changes in the respective tumors at each time-point after MNP administration was calculated relative to that before MNP administration, using black pixel analysis (Supplementary Material) 52, 56-58. This method has been reported to measure high doses of MNPs in biological systems effectively 52, 56-58. Thus, since high doses of MNPs were used in this study (50 mg/kg), this method was used to quantify the relative amounts of MNPs delivered to the tumors.

### Histology

Histology was used to demonstrate that at the tumor sizes (~50 mm^3^) used in the current study, necrosis was not detectable at the tumor cores. Tumors (~50 mm^3^) were harvested from non-treated tumor-bearing mice (not included in Scheme 1) and fixed in 10% neutral buffered formalin for 24 h. The tumors were then paraffin-embedded and sectioned into 30 µm slices. Tumor sections were next stained with hematoxylin and eosin (H & E), using a standard protocol. Bright-field optical microscopy images were then acquired using an Aperio ScanScope^®^ AT system (Vista, CA). The images were next analyzed using NIH ImageJ software for any signs of necrosis at the tumor cores.

### Multi-photon microscopy

Two-photon microscopy was used to study the vascular distribution patterns in tumors of the approximate sizes (~50 mm^3^) used in the current study. It was also used to elucidate how intra-tumoral vascular distribution patterns affected MNP delivery. Tumor-bearing mice (not included in Scheme 1) were intravenously administered 50 µL of a 2,000 kDa Texas Red conjugated dextran polymer (Sigma Aldrich), prepared at 1 µg/µL, as previously described 52. The mice were then euthanized, and the tumors were excised immediately and sectioned into 1 mm slices. Each tumor slice was classified into two main regions as previously reported: 1) The tumor periphery (< 2 mm from the tumor margin) and 2) the tumor center (˃ 2 mm from the tumor margin) 52. Tumor sections were then imaged immediately after sectioning on an Olympus Laser Scanning FV1000 MPE microscope at λ_ex_ = 820 nm and λ_em_ = 615 nm. At least three fields of view (FOVs) were acquired from each of the two regions on each tumor slice. A 25× objective was used to acquire confocal z-stacks with FOV 508 × 508 μm^2^, and z-intervals of 5 μm. All images were analyzed using NIH ImageJ software.

### Statistical analyses

Data were presented as the mean ± standard deviation of at least three independent experiments (technical replicates). Statistical analyses were performed using paired two-tailed t-tests. All results were considered statistically significant at *P* < 0.05, except when otherwise stated.

## Results

### *In vivo* fluorescence image-guided PDT

**Figure 2:**
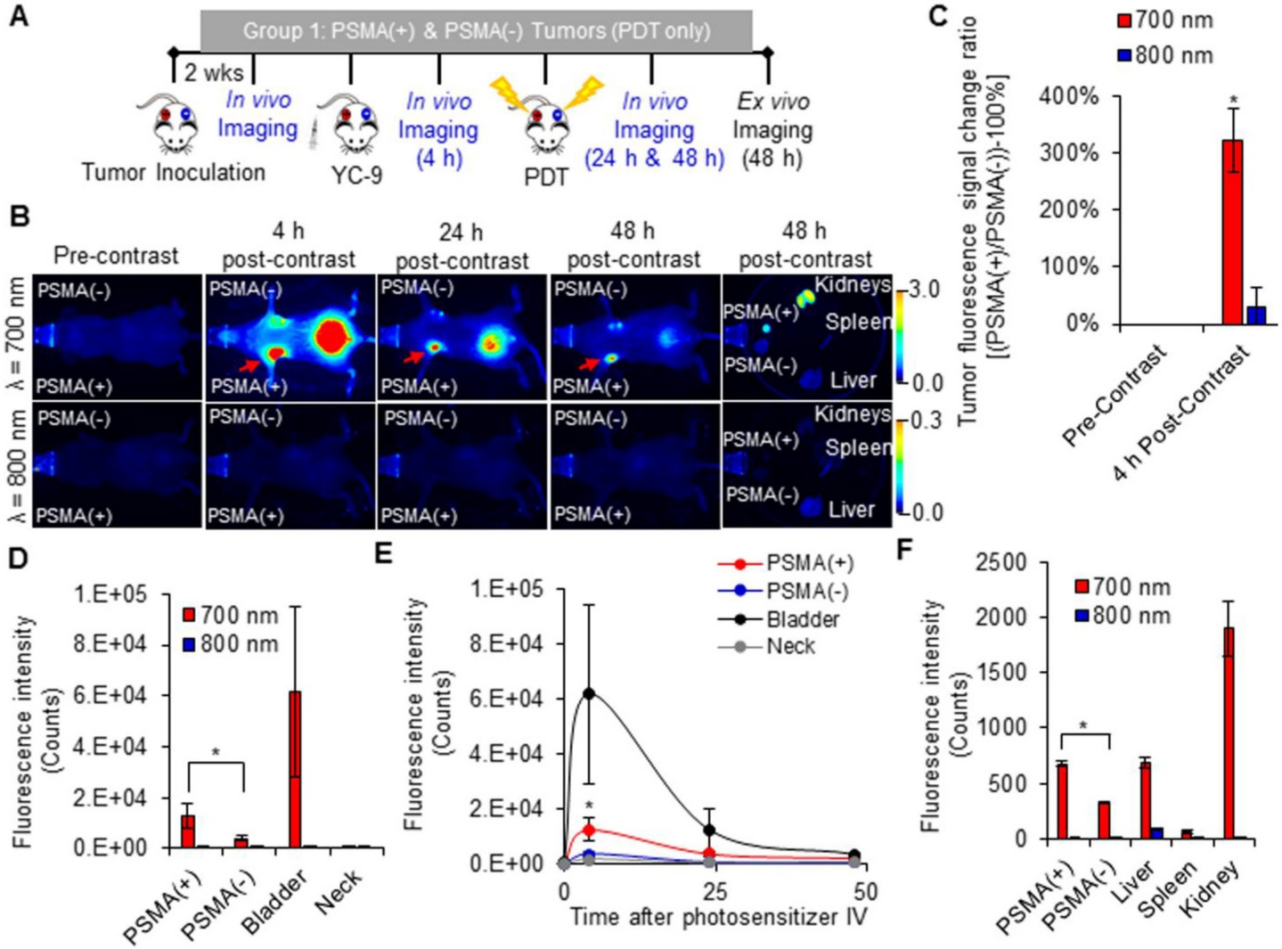
*In vivo* fluorescence image-guided PDT. **A)** Schematic representing the experimental design for Group 1 mice. **B)** 700 nm and 800 nm* in vivo* fluorescence images of a representative male NSG mouse bearing both human PSMA(+) PC3 PIP and PSMA(-) PC3 flu tumor xenografts, 0 h, 4 h, 24 h, and 48 h after the intravenous administration of YC-9. **C)** Quantification of the fluorescence change ratios of PSMA(+) tumors to PSMA(-) tumors within each mouse, 0 h and 4 h after YC-9 administration, from the 700 nm and 800 nm fluorescence signals, respectively (*P* = 0.015; n = 3). **D)** Quantification of the 700 nm *in vivo* fluorescence signal, 4 h after YC-9 administration (*P* = 0.023; n = 3). **E)** *In vivo* clearance of YC-9 from different organs of the mouse over 48 h (*P* = 0.023; n = 3). **F)** Quantification of the 700 nm *ex vivo* fluorescence signal, 48 h after YC-9 administration and 42 h after PDT (*P* = 0.003; n = 3).

An increase in the fluorescence signal was detected only in the 700 nm channel at all time-points after YC-9 administration in the Group 1 mice (Figure 2 and Figure S2), as expected. The fluorescence signal ratio of the PSMA(+) tumor compared to the PSMA(-) tumor within each mouse, 4 h after YC-9 administration was compared to that before YC-9 administration, using both the 700 nm and 800 nm fluorescence signals, respectively (Figure 2C). A significant (*P* = 0.023; n = 3) difference in the accumulation of YC-9 was detected in PSMA(+) tumors compared to PSMA(-) tumors, 4 h post-YC-9 administration. This is in accordance with our previous report 50. At the 4 h time-point, YC-9 accumulated 3.2 ± 0.7 times more in PSMA(+) tumors than in the PSMA(-) tumors and 16.6 ± 7.9 times more in PSMA(+) tumors than in other regions of the mouse body, such as the neck (Figure 2D). However, significant uptake of YC-9 was detected in both PSMA(+) and PSMA(-) tumors 4 h after YC-9 administration compared to that at the 0 h time-point, in the 700 nm channel (*P* = 0.015; n = 3 and *P* = 0.011; n = 3, respectively). A high concentration of YC-9 was also detected in the bladder at this time-point, suggesting some renal clearance.

The signal from YC-9 in the different organs of the mice decreased over the next 42 h post-PDT (Figure 2E), suggesting further YC-9 clearance from the body. However, a significant difference (*P* = 0.003; n = 3) in the uptake of YC-9 was still detected *ex vivo* in PSMA(+) tumors compared to PSMA(-) tumors, 42 h after PDT (Figure 2F).

### *In vivo* MRI of early tumor response to PDT

**Figure 3:**
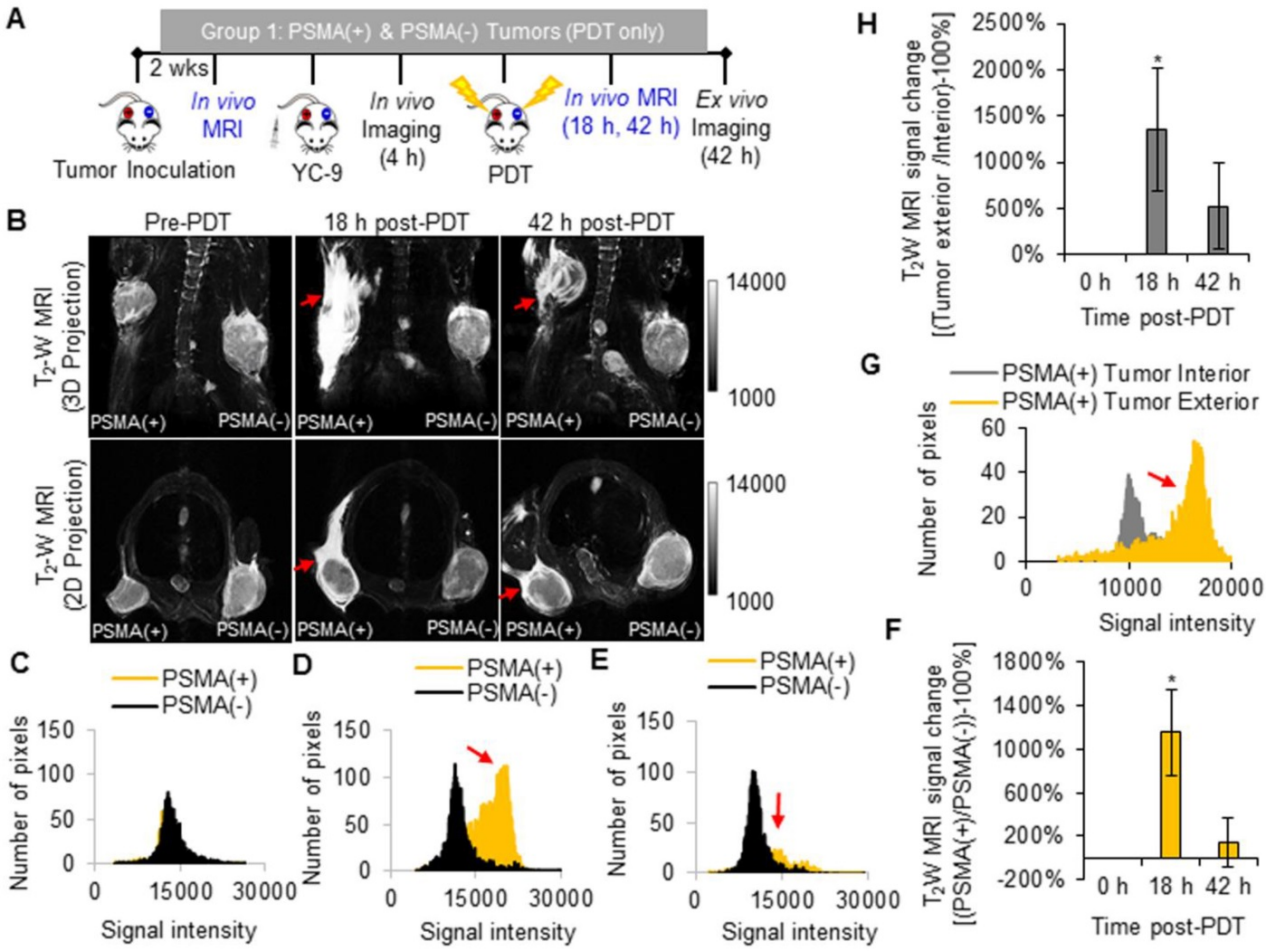
*In vivo* T_2_ -W MRI of edema after PDT. **A)** Schematic representing the experimental design for Group 1 mice. **B)** *In vivo* T_2_ -W MRIs [coronal view (top row) and axial view (bottom row)], of a representative male NSG mouse bearing human PSMA(+) PC3 PIP and PSMA(-) PC3 flu tumor xenografts, 0 h, 18 h, and 42 h after PDT. Pixel intensity histograms of PSMA(+) PC3 PIP and PSMA(-) PC3 flu tumor xenografts, **C)** before PDT; **D)** 18 h after PDT; and **E)** 42 h after PDT. **F)** T_2_W MRI signal change ratios of PSMA(+) PC3 PIP tumors compared to PSMA(-) tumors, 0 h, 18 h and 42 h after PDT (*P* = 0.007; n = 3). **G)** Pixel intensity histogram of PSMA(+) PC3 PIP tumor exterior versus tumor interior. **H)** The T_2_W MRI signal change ratio of the PSMA(+) PC3 PIP tumor exterior versus the tumor interior indicates greater edema in the tumor exterior compared to the tumor interior, 18 h and 42 h post-PDT (*P* = 0.024; n = 3).

T_2_ -W MRI was used to identify markers of early tumor response to PDT. Tumor response markers evaluated included: edema markers, identified as hyperintensity on T_2_ -W MRIs of the PDT-treated areas; and vascular damage or hemorrhage markers, identified as hypointensity on T_2_ -W MRIs of the PDT-treated areas. T_2_ -W MRI and the above-mentioned MRI markers were chosen since they do not require the intravenous administration of a contrast agent, which could interfere with the MRI signal from MNPs after delivery. Group 1 mice were imaged with T_2_ -W MRI before PDT and 18 h and 42 h after PDT (Figure 3A).

Significant edema was detected in the tissue surrounding the PSMA(+) tumors compared to that surrounding the PSMA(-) tumors (*P* = 0.007; n = 3) at 18 h after PDT (Figure 3B). This edema in the tissue surrounding the PSMA(+) tumor decreased by the 42 h post-PDT time-point. Bright pixel analysis, previously reported and described in the Supplementary Material (Method S3, S4, and Figures S3 and S4), was used to quantify edematous changes in the PSMA(+) tumor surroundings compared to that of the PSMA(-) tumors within each mouse, before and after PDT 56. Briefly, a shift in the pixel distribution towards higher intensity pixels was detected 18 h after PDT (Figure 3C-E). By quantifying changes in high intensity pixels using bright pixel analysis, the T_2_-W MRI signal change ratios of PSMA(+) tumor surroundings, compared to PSMA(-) tumors surroundings in each mouse, could be calculated after PDT. An 11.5 ± 3.9 fold increase in edema was detected in the PSMA(+) tumor surroundings compared to the PSMA(-) tumor surroundings, 18 h after PDT versus before PDT (Figure 3F). That T_2_-W MRI signal ratio decreased to 1.4 ± 2.2 fold by the 42 h post-PDT time-point (Figure 3F). Additionally, 13.6 ± 6.6 fold more edema was detected in the PSMA(+) tumor surroundings compared to the PSMA(+) tumor interiors at 18 h after PDT (Figure 3G-H). That difference in edema in the PSMA(+) tumor surroundings compared to the PSMA(-) tumor surroundings was attributed to the 3.2 ± 0.7 fold higher accumulation of the PSMA-targeted photosensitizer (YC-9) in PSMA(+) tumors compared to PSMA(-) tumors, before NIR irradiation for PDT.

**Figure 4:**
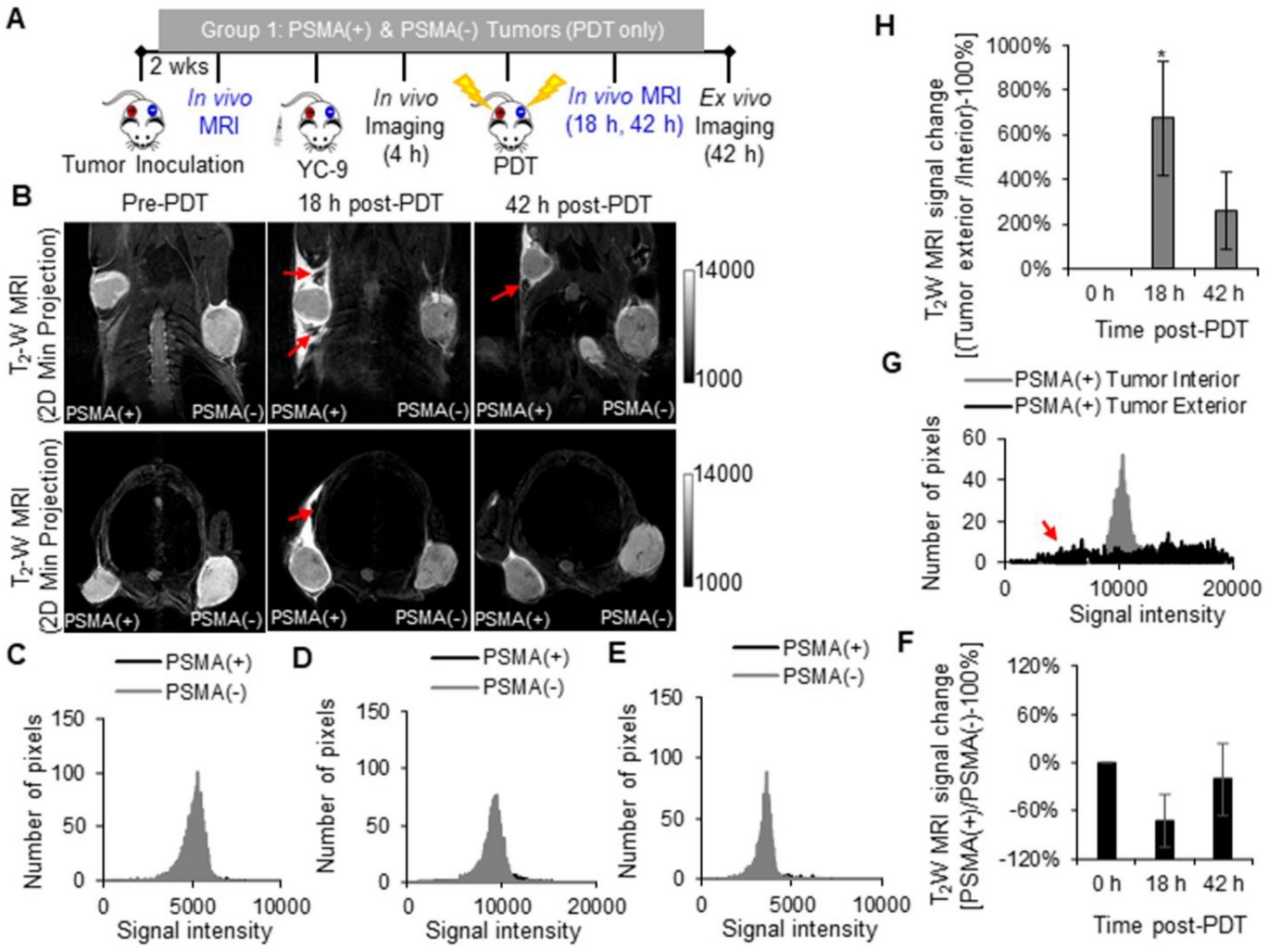
*In vivo* T_2_ -W MRI of hemorrhage in tumor surroundings after PDT. **A)** Schematic representing the experimental design for Group 1 mice. **B)** *In vivo* T_2_ -W MRI, 2D Z-projections (minimum) of a representative male NSG mouse bearing human PSMA(+) PC3 PIP and human PSMA(-) PC3 flu tumor xenografts, 0 h, 18 h, and 42 h after PDT [coronal view (top row) and axial view (bottom row)]. Pixel intensity histograms of PSMA(+) PC3 PIP and PSMA(-) PC3 flu tumor interiors, **C)** before PDT; **D)** 18 h after PDT; and **E)** 42 h after PDT. **F)** T_2_W MRI signal change ratios of PSMA(+) PC3 PIP tumor interiors compared to PSMA(-) PC3 flu tumor interiors 0 h, 18 h, and 42 h after PDT. **G)** Pixel intensity histogram of a PSMA(+) PC3 PIP tumor exterior versus tumor interior. **H)** The T_2_W MRI signal change ratio of the PSMA(+) PC3 PIP tumor exterior versus the tumor interior (*P* = 0.010; n = 3) indicates hemorrhage in the tumor exterior but not in the tumor interior.

In addition to edema, hemorrhage was also detected in the PSMA(+) tumor surroundings compared to the PSMA(-) tumor surroundings 18 h after PDT (Figure 4). That hemorrhage in the PSMA(+) tumor surroundings decreased by the 42 h post-PDT time-point (Figure 4). However, no significant hemorrhage was detected in the PSMA(+) tumor interiors compared to PSMA(-) tumor interiors 18 h and 42 h after PDT, when compared to that before PDT (Figure 4B). To quantify hemorrhage in the PDT-treated areas, black pixel analysis was used to quantify hypointensity changes in PSMA(+) tumors compared to PSMA(-) tumors in each mouse, before and after PDT (Figure 4C-F and Supplementary Method S5 and Figure S5) 52, 56. Black pixel analysis was also used to quantify hypointensity changes in the exterior versus the interior of PSMA(+) tumors at 18 h and 42 h after PDT (Figure 4G-H and Figure S6). These results collectively suggested that T_2_-W MRI could be used to detect MNP delivery to tumors after PDT-mediated enhanced tumor vascular permeability since no significant hemorrhage was seen within either of the tumors 18 h and 42 h post-PDT.

### *In vivo* fluorescence imaging of enhanced MNP delivery after PDT

**Figure 5:**
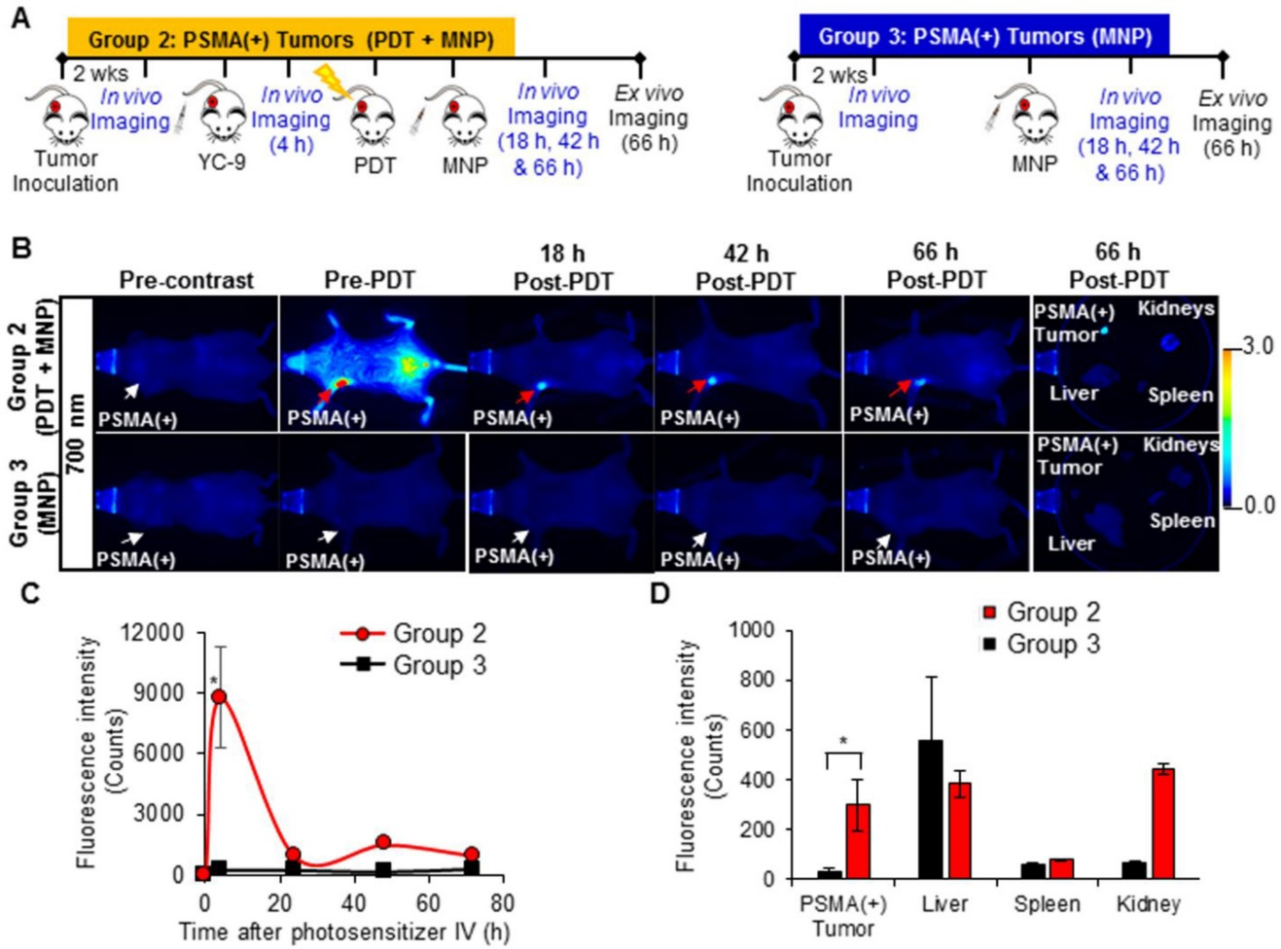
*In vivo* fluorescence image-guided PDT in PSMA(+) tumor-bearing mice. **A)** Schematic representing the experimental design for Groups 2 and 3 mice, respectively. **B)** 700 nm *in vivo* fluorescence images of representative male NSG mice bearing human PSMA(+) PC3 PIP tumor xenografts, from Group 2 and Group 3, respectively. **C)** Quantification of the 700 nm *in vivo* fluorescence signal in PSMA(+) tumors of Group 2 mice compared to those from Group 3 mice (*P* = 0.023; n = 3), over 72 h post-YC-9 administration (66 h post-PDT). **D)** Quantification of the 700 nm *ex vivo* fluorescence signal from the organs of Group 2 mice compared to those from Group 3 mice (*P* = 0.008; n = 3), 72 h after YC-9 administration (66 h post-PDT).

A significant increase in the 700 nm *in vivo* fluorescence signal was detected in the PSMA(+) tumors of Group 2 mice (that received YC-9), compared to Group 3 mice (that did not receive YC-9) (*P* = 0.023; n = 3), 4 h post-YC-9 administration (pre-PDT in Figure 5B and Figure S7). YC-9 was also detected in the bladders of Group 2 mice at this time-point, suggesting some renal clearance of the photosensitizer. The signal from YC-9 in the different organs of the Group 2 mice decreased over the next 72 h post-YC-9 administration (66 h post-PDT), suggesting additional YC-9 clearance (Figure 5B-C). However, a significant difference (*P* = 0.008; n = 3) in the uptake of YC-9 was still detected *ex vivo* in PSMA(+) tumors of Group 2 mice compared to those of Group 3 mice (Figure 5D), 72 h after YC-9 administration (66 h post-PDT).

A significant increase in the 700 nm *in vivo* fluorescence signal from YC-9 was also detected in the PSMA(-) tumors of Group 4 mice (that received YC-9), compared to Group 5 mice (that did not receive YC-9), 4 h post-YC-9 administration (*P* = 0.017, n = 3, Figure S8 and Figure S9). However, the fluorescence signal in the PSMA(+) tumors of Group 2 mice was ~3.5-fold higher than that detected in the PSMA(-) tumors of Group 4 mice.

**Figure 6:**
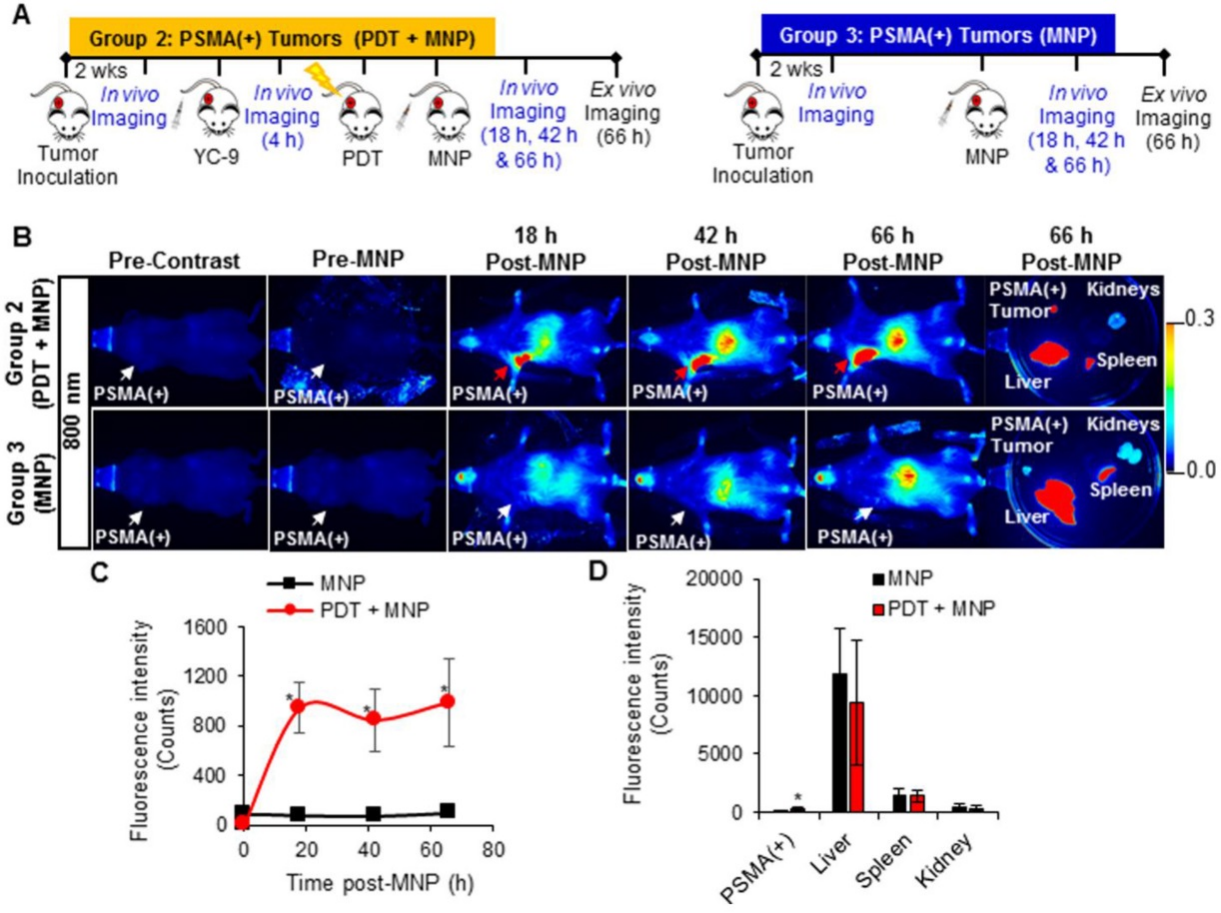
*In vivo* fluorescence imaging of enhanced MNP delivery to PSMA(+) tumors, after PSMA-targeted PDT. **A)** Schematic representing the experimental design for Groups 2 and 3 mice, respectively. **B)** 800 nm *in vivo* fluorescence images of representative male NSG mice bearing human PSMA(+) PC3 PIP tumor xenografts, from Group 2 and Group 3, respectively. Group 2 mice were treated with PDT before the administration of the MNPs, while Group 3 mice were not treated with PDT. **C)** Quantification of the 800 nm *in vivo* fluorescence signal from the PSMA-targeted MNPs in PSMA(+) tumors of Group 2 mice compared to Group 3 mice, over 66 h post-MNP administration (*P* ≤ 0.019; n = 3). **D)** Quantification of the PSMA-targeted MNPs in the organs of Group 2 mice compared to those of Group 3 mice, 66 h after MNP administration (*P* ≤ 0.031; n = 3).

A significant increase (*P* ≤ 0.019; n = 3) in the uptake of PSMA-targeted MNPs was also detected *in vivo*, in the PSMA(+) tumors of Group 2 mice compared to Group 3 mice, 18 h, 42 h, and 66 h post-PDT and MNP administration, in the 800 nm channel (Figure 6 and Figure S7). Quantification of the 800 nm *in vivo* fluorescence signal revealed: 11.9-fold; 11.5-fold; and 9.9-fold higher accumulation of the PSMA-targeted MNPs in the PSMA(+) tumors of Group 2 mice compared to Group 3 mice, 18 h, 42 h, and 66 h post-PDT and MNP administration, respectively. Statistically (*P* ≤ 0.031; n = 3), more of the PSMA-targeted MNPs were also detected *ex vivo* in the PSMA(+) tumors of Group 2 mice compared to Group 3 mice, 66 h post-MNP administration (Figure 6E).

Using the 800 nm *ex vivo* fluorescence signal to determine the organ distribution ratio, 66 h post-MNP administration, we calculated the following percentage of the injected dose (% ID) in the organs of Group 2 mice: 2.8 ± 0.6% ID of the administered PSMA-targeted MNPs were present in the PSMA(+) tumors; 79.8 ± 4.0% ID in the liver; 14.4 ± 2.2% ID in the spleen; and 3.0 ± 1.4% ID in the kidney (Table S2). On the other hand, in Group 3 mice not treated with PDT, we calculated: 0.6 ± 0.4% ID of the administered PSMA-targeted MNPs were present in the PSMA(+) tumors; and 86.3 ± 1.0% ID in the liver; 11.2 ± 1.2% ID in the spleen; and 1.9 ± 0.4% ID in the kidney (Table S3).

A significant increase (*P* ≤ 0.018; n = 3) in the uptake of the PSMA-targeted MNPs was also detected *in vivo,* in the PSMA(-) tumors of Group 4 mice (pretreated with PDT) compared to Group 5 mice (not treated with PDT), 18 h, 42 h, and 66 h post-PDT and MNP administration, on the 800 nm channel (Figure S9 and Figure S10). Quantification of the 800 nm *in vivo* fluorescence signal revealed: 5.4-fold; 5.6-fold; 3.6-fold, higher accumulation of the PSMA-targeted MNPs in the PSMA(-) tumors of Group 4 mice compared to Group 5 mice, 18 h, 42 h, and 66 h post-PDT and MNP administration, respectively. A significant difference (*P* = 0.020; n = 3) was also detected between both groups, *ex vivo* (Figure S8E).

Using the *ex vivo* fluorescence signal to determine the organ biodistribution ratio, 66 h post-MNP administration, in Group 4 mice, we calculated the following: 5.7 ± 1.3% ID of the administered PSMA-targeted MNPs were present in the PSMA(-) tumors; 82.4 ± 4.8% ID in the liver; 9.3 ± 3.4% ID in the spleen; and 2.6 ± 2.0% ID in the kidney (Table S4). On the other hand, in Group 5 mice not treated with PDT, we calculated 2.7 ± 0.4% ID of the administered PSMA-targeted MNPs were present in the PSMA(-) tumors; and 83.6 ± 3.3% ID in the liver; 11.6 ± 2.8% ID in the spleen; and 2.1 ± 0.1% ID in the kidney (Table S5).

### *In vivo* MRI of enhanced MNP delivery after PDT

**Figure 7:**
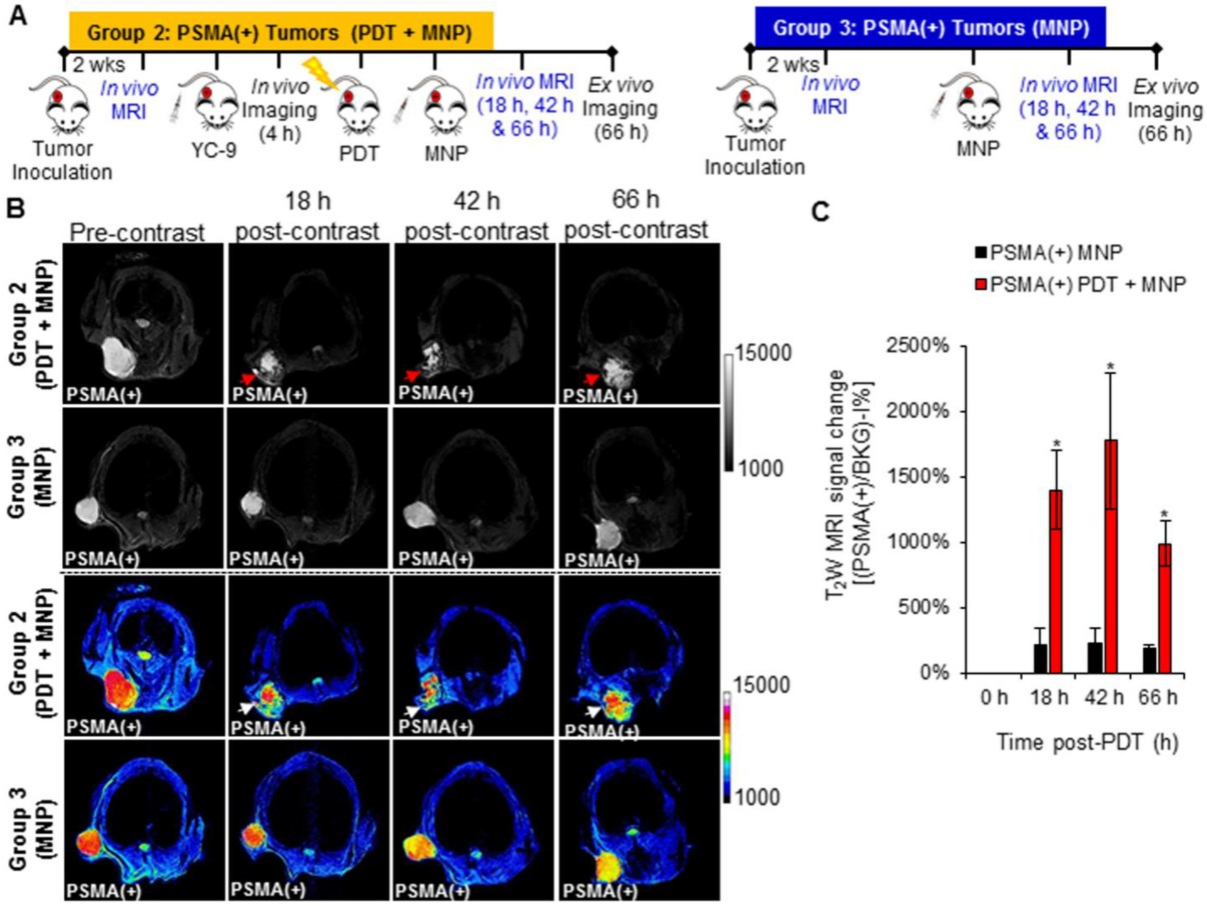
*In vivo* MRI of enhanced MNP delivery to PSMA(+) tumors, after PSMA-targeted PDT. **A)** Schematic representing the experimental design for Groups 2 and 3 mice, respectively. **B)** *In vivo* MRI of representative male NSG mice bearing human PSMA(+) PC3 PIP tumor xenografts, from Group 2 and Group 3, respectively, 0 h, 18 h, 42 h, and 66 h after the administration of PSMA-targeted MNPs. **C)** T_2_W MRI signal change ratios of PSMA(+) PC3 PIP tumors in Group 2 mice compared to those of PSMA(+) PC3 PIP tumors in Group 3 mice, 0 h, 18 h, 42 h, and 66 h after MNP administration (*P* ≤ 0.008; n = 3).

A significant increase (*P* ≤ 0.008; n = 3) in the uptake of PSMA-targeted MNPs was detected in the PSMA(+) tumors of Group 2 mice (pretreated with PDT) compared to those of Group 3 mice (not treated with PDT), 18 h, 42 h, and 66 h post-PDT and MNP administration, using T_2_-W MRI (Figure 7B). A black pixel analysis derivative adapted for single tumor mouse models (Method S6 and Figures S11) was used to quantify the accumulation of the PSMA-targeted MNPs in PSMA(+) tumors of Group 2 mice compared to those of Group 3 mice, 18 h, 42 h, and 66 h post-PDT. A prominent left shift towards lower intensity pixels was detected in the tumor pixel intensity distribution histogram, 18 h, 42 h, and 66 h after the administration of the PSMA-targeted MNPs in Group 2 mice but not in Group 3 mice (Figure S12). Quantification revealed: 6.7-fold; 7.7-fold; and 5.5-fold more of the PSMA-targeted MNPs accumulated in the PSMA(+) tumors of Group 2 mice compared to those of Group 3 mice, 18 h, 42 h, and 66 h post MNP administration (Figure 7C).

A significant difference (*P* ≤ 0.018; n = 3) in the uptake of PSMA-targeted MNPs was also detected between PSMA(-) tumor-bearing Group 4 mice (pretreated with PDT) and Group 5 mice (not treated with PDT), 18 h, and 42 h post-MNP administration, using T_2_-W MRI (Figure S13 and S14). No statistically significant difference was detected between both groups 66 h post-MNP administration. Quantification revealed: 2.9-fold, 2.6-fold, and 1.7-fold higher accumulation of the PSMA-targeted MNPs in the PSMA(-) tumors of Group 4 mice compared to Group 5 mice, 18 h, 42 h, and 66 h post-MNP administration. The lower difference detected with T_2_-W MRI compared to fluorescence imaging between the PDT pretreated groups and the non-treated groups was attributed to the lower sensitivity of MRI compared to fluorescence imaging. However, the MRI values were comparable to those obtained with fluorescence imaging.

### Imaging the effects of tumor vasculature on MNP delivery

**Figure 8:**
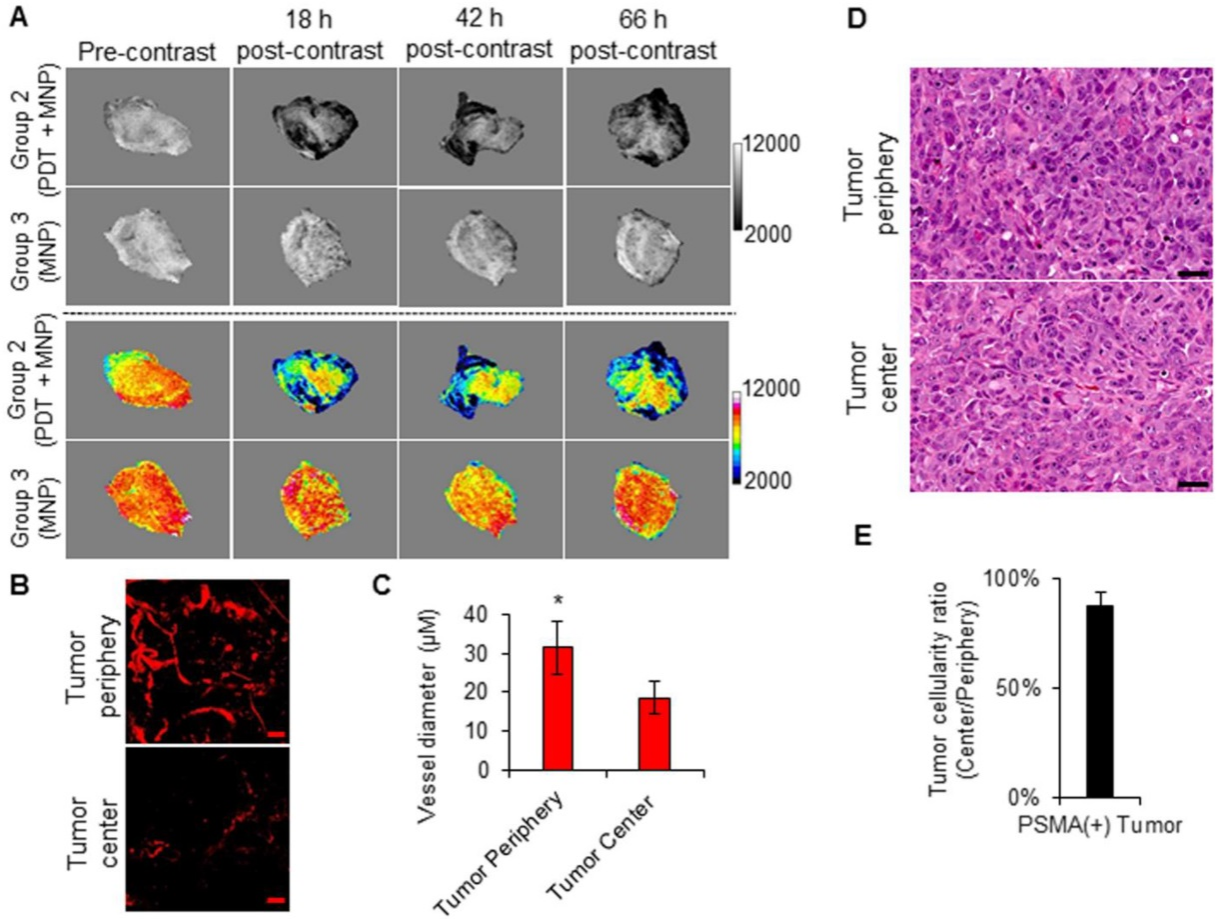
Imaging intra-tumoral MNP and vascular distribution patterns. **A)** T_2_-W MRIs (grayscale and colored) of the intra-tumoral signal change patterns of representative PSMA(+) PC3 PIP tumors from Group 2 and Group 3 mice, respectively. The signal change patterns were indicative of the intra-tumoral distribution of the delivered PSMA-targeted MNPs. **B)** Two-photon fluorescence microscopy images of human PSMA(+) PC3 PIP tumors, excised from mice after the intravenous administration of a 2,000 kDa Texas Red conjugated dextran polymer. The images show a higher vascular density at the tumor periphery compared to the tumor center. The scale bar represents 50 µm. **C)** Quantification of tumor blood vessel diameters at the tumor peripheries and the tumor centers. Blood vessels of larger diameters were found at the tumor periphery compared to the tumor center (*P* = 0.008; n = 3). **D)** Hematoxylin and eosin (H&E) staining of the tumor center compared to the tumor periphery of human PSMA(+) PC3 PIP prostate tumors, excised from non-treated mice. The scale bar represents 50 µm. **E)** Quantification of the ratio of loss of cellularity at the tumor center compared to the tumor periphery. This revealed no significant necrosis at either the tumor peripheries or the tumor centers.

Using the high spatial resolution of MRI, we assessed the intra-tumoral distribution of the delivered PSMA-targeted MNPs in the PSMA(+) tumors of Group 2 and Group 3 mice, respectively. The T_2_-W MRIs revealed preferential signal changes at the tumor peripheries instead of homogenous signal changes throughout the tumors (Figure 8A). A similar signal change pattern was detected in the PSMA(-) tumors of Group 4 and Group 5 mice (Figure S15A). This finding is in accordance with our previous reports 51, 52.

By studying the intra-tumoral vasculature distribution patterns of PSMA(+) tumors and PSMA(-) tumors at the approximate sizes (~50 µm) used in the current study, using multi-photon microscopy, we detected higher vascular densities at the tumor peripheries compared to the tumor centers (Figure 8B and Figure S15B). Blood vessels of larger diameters were also detected at the tumor peripheries compared to the tumor centers (Figure 8C and Figure S15C). Finally, H&E staining revealed no differences in tissue cellularity and subsequently no differences in tissue necrosis between the tumor peripheries and the tumor centers of PSMA(+) tumors of the approximate sizes (~50 µm) used in the current study (Figure 8C-D, Figure S15C-D, and S16). This suggested that the difference in the intra-tumoral vascular distribution was not the result of tumor necrosis. Collectively, these results indicated that the intra-tumoral vascular density and distribution pattern influenced the intra-tumoral MNP delivery and distribution pattern post-PDT.

## Discussion

MNP-induced hyperthermia is currently being evaluated as a less morbid focal therapy for intermediate and high-risk localized prostate cancers 6, 7, 10. However, since the delivery of high concentrations of MNPs to tumors after intravenous administration is still a major challenge, current preclinical and clinical practices involve directly injecting the MNPs into tumors 6, 59. Direct injection can produce treatment-related morbidity, which can negatively impact the quality of life of patients post-treatment, and will miss metastatic foci 10.

Previously, we evaluated the feasibility of specifically enhancing the delivery of a PSMA-targeted MNP to PSMA(+) tumors in a preclinical dual PSMA(+) and PSMA(-) human prostate tumor mouse model, with high tumor vascular permeability 52. Tumor vascular permeability was modulated in that study by using large tumors (~250 mm^3^). In that study, we observed that although administering high MNP doses [50 mg/kg (30 mg of Fe/kg)] increased the amount of MNPs that accumulated in PSMA(+) tumors, it also increased the concentration of the MNPs that accumulated non-specifically in PSMA(-) tumors and the organs of the reticuloendothelial system (RES).

In this report, we evaluated a different strategy to enhance specifically the delivery of PSMA-targeted MNPs to PC tumors. We used our previously developed low-molecular-weight PSMA-targeted photosensitizer (YC-9) and a PDT pretreatment plan to enhance the vascular permeability of PSMA(+) tumors, with low tumor vascular permeability (~50 mm^3^), for the increased delivery of our previously developed PSMA-targeted MNPs [50 mg/kg (30 mg of Fe/kg)].

To estimate the contribution from the baseline tumor vascular permeability on MNP delivery in the PSMA(+) and PSMA(-) tumors, respectively, we estimated MNP delivery to the untreated PSMA(+) tumors (Group 3) and the untreated PSMA(-) tumors (Group 5), respectively. Using* ex vivo* fluorescence imaging (Table S3 and S5), we observed that the delivery of MNPs to the untreated PSMA(-) tumors (Group 5) was ~ 4.5-fold higher than to the untreated PSMA(+) tumors (Group 3). This suggested a higher contribution from the baseline vascular permeability in the PSMA(-) tumors compared to the PSMA(+) tumors. This difference was attributed to larger volumes of the PSMA(-) tumors than the PSMA(+) tumors. Thus, to take into account this baseline difference in the vascular permeability between the PSMA(+) and the PSMA(-) tumors in this study, direct comparisons were not made between the PDT pretreated PSMA(+), and the PDT pretreated PSMA(-) tumors. Instead, an indirect comparison was made as described below.

To evaluate the non-specific contributions from non-targeted PDT to enhance MNP delivery, tumors from PDT pretreated PSMA(-) PC3 flu (Group 4) mice were compared to those from untreated PSMA(-) PC3 flu (Group 5) mice. *In vivo* fluorescence and MRI results suggested a 3.6 ± 1.6-fold higher delivery of the MNPs in the PDT pretreated PSMA(-) tumors (Group 4), compared to the untreated PSMA(-) tumors (Group 5).

The contribution from PSMA-targeted PDT to enhance MNP delivery to tumors was next indirectly evaluated by comparing the difference between the PSMA(+) PC3 PIP groups versus that between the PSMA(-) PC3 flu groups [(Group 2 versus Group 3) compared to (Group 4 versus Group 5)]. From the* in vivo* fluorescence and MRI results, a 8.9 ± 2.6-fold higher delivery of the MNPs was detected in the PDT pretreated PSMA(+) tumors (Group 2), compared to the untreated PSMA(+) tumors (Group 3). Thus, by comparing the difference between the PSMA(+) groups to the difference between the PSMA(-) groups, the ~ 2-fold higher difference detected between the PSMA(+) groups was attributed to the specific contribution from PSMA-targeted PDT. These EPR effect enhancement values are comparable to those observed with other previously reported agents, designed for different tumor phenotypes 33, 42, 60.

Using the *ex vivo* fluorescence organ biodistribution ratios, we estimated that ~0.34 mg of iron/cm^3^ of the tumor volume was deposited in the PDT pretreated PSMA(+) tumors versus ~0.07 mg of iron/cm^3^ of the tumor volume in the non-treated PSMA(+) tumors. That iron concentration determined in the PDT-pretreated PSMA(+) tumors is higher than that reported to be required for efficient hyperthermia therapy (0.27 mg of iron/cm^3^ of the tumor volume), following direct tumor injections in rodents 59. However, the intra-tumoral MNP distribution was inhomogeneous. This could leave some tumor areas untreated and subsequently result in tumor regrowth and patient relapse 61, 62. Consequently, several strategies are currently being developed to homogenously enhance tumor vascular permeability and the delivery of MNPs to tumors 26, 61, 62. Furthermore, although this PDT pretreatment strategy was effective in enhancing the delivery of PSMA-targeted MNPs to PSMA(+) tumors with low tumor vascular permeability, there is still a need for the development and optimization of MNP delivery strategies to increase the delivery efficiency, by minimizing the accumulation of MNPs in organs of the RES, such as the liver.

## Conclusion

We evaluated the feasibility of increasing the delivery of PSMA-targeted MNPs to PSMA(+) tumors with low tumor vascular permeability. Through the use of two complementary imaging techniques, we demonstrated that the delivery of PSMA-targeted MNPs to PSMA(+) tumors could be enhanced by PSMA-targeted PDT using a low-molecular-weight PSMA-targeted photosensitizer via the enhancement of tumor vascular permeability. MNP-induced hyperthermia and vascular-targeted PDT are both focal therapies currently being evaluated for the treatment of localized PC. Consequently, the use of low dose PSMA-targeted PDT to enhance the delivery of PSMA-targeted MNPs could contribute synergistically to effective long-term control of aggressive localized PC lesions. We anticipate that this strategy could be used to deliver drug-loaded, PSMA-targeted MNPs to localized, aggressive, PSMA-expressing, castration-resistant prostate tumors for enhanced MRI/MPI-guided hyperthermia, and sustained drug release.

## Supplementary Material

Supplementary materials, figures and tables.

## Abbreviations

EPR: enhanced permeability and retention
FBS: fetal bovine serum
FOV: field of view
H&E: hematoxylin and eosin
ID: injected dose
LED: light-emitting diode
MNP: magnetic nanoparticle
MPE: multi-photon excitation
MPI: magnetic particle imaging
MRI: magnetic resonance imaging
MS: matrix size
NA: number of averages
NHS: *N*-hydroxy succinimide
NIR: near infra-red
NIH: National Institute of Health
NSG: non-obese diabetic severe-combined immunodeficient gamma
PC: prostate cancer
PDT: photodynamic therapy
PEG: polyethylene glycol
PIT: photoimmunotherapy
PSMA: prostate-specific membrane antigen
PSMA(-): prostate-specific membrane antigen negative
PSMA(+): Prostate-specific membrane antigen positive
RARE: rapid acquisition with refocused echoes
RES: reticuloendothelial system
ST: slice thickness
TE: echo time
TE_Eff_: effective echo time
T_2_-W: T_2_-weighted
TR: repetition time
YC-9: PSMA-targeted photosensitizer
