# Supplementary material for: Prostate-specific membrane antigen (PSMA)-targeted photodynamic therapy enhances the delivery of PSMA-targeted magnetic nanoparticles to PSMA-expressing prostate tumors
Source: Nanotheranostics. 2021 Jan 19;5(2):182–96. doi: 10.7150/ntno.52361 (PMC7868004; doi:10.7150/ntno.52361)
Supplement: Supplementary file 1 — Supplementary materials, figures and tables. [file ntnov05p0182s1.pdf]

Prostate-specific membrane antigen (PSMA)-targeted photodynamic therapy enhances the delivery of PSMA-targeted magnetic nanoparticles to PSMA expressing prostate tumors

Ethel J. Ngen[1], Ying Chen[1], Babak Behnam Azad[1], Srikanth Boinapally[1], Desmond Jacob[1], Ala Lisok[1], Chentian Shen[1], Mir S. Hossain[1], Jiefu Jin[1], Zaver M. Bhujwalla[1,2], Martin G. Pomper[1,2], Sangeeta R. Banerjee[1,2,3*]

[1] The Russell H. Morgan Department of Radiology and Radiological Science, Johns Hopkins University School of Medicine, Baltimore, MD 21287, USA.

[2] The Sidney Kimmel Comprehensive Cancer Center, Johns Hopkins University School of Medicine, Baltimore, MD 21231, USA.

[3] The F. M. Kirby Research Center for Functional Brain Imaging, Kennedy Krieger Research Institute, Baltimore, Maryland 21205, USA.

[*]Correspondence should be addressed to: Sangeeta R. Banerjee, Ph.D. (sray9@jhmi.edu)

Supplementary Method

Method S1 -- 4

Method S2 -- 4

Method S3 -- 6

Method S4 -- 7

Method S5 -- 8

Method S6 -- 9

Supplementary Figures

Figure S1 -- 10

Figure S2 -- 10

Figure S3 -- 11

Figure S4 -- 12

Figure S5 -- 13

Figure S6 -- 14

Figure S7 -- 15

Figure S8 -- 16

Figure S9 -- 17

Figure S10 -- 18

Figure S11 -- 19

Figure S12 -- 20

Figure S13 -- 21

Figure S14 -- 22

Figure S15 -- 23

Figure S16 -- 24

Supplementary Tables

Table S1 --- 25

Table S2 --- 25

Table S3 --- 25

Table S4 --- 25

Table S5 --- 25

Supplementary Methods

S1. Characterization of PSMA-targeted MNPs.

PSMA-targeted MNPs were successfully functionalized, as previously reported.[1, 2] The hydrodynamic diameter (Z-average), polydispersity index (PDI), and the ζ-potential of the PSMA-targeted MNPs were measured using a Malvern ζ-sizer Nano ZS-90. For size measurements, the MNPs were suspended in water at 10 µg/mL, and both the hydrodynamic diameter and the PDI were measured at 25 °C, using disposable square polystyrene cuvettes. The hydrodynamic diameter and PDI results were represented as the average value of three measurements, with each measurement consisting of 30 runs. The ζ-potentials of the MNPs suspended in water (10 µg/mL) were next measured using disposable capillary cells. The results were represented as the average value of three measurements, with 20 - 25 runs within each measurement.

PSMA-targeted MNPs possessed a hydrodynamic diameter of 147 ± 8 nm and a ζ-potential of -10.9 ± 0.3 mV (Table S1).[2] These values were different from those obtained from the unmodified MNPs, with a hydrodynamic diameter of 133 ± 1 nm and a ζ-potential of +24.0 ± 2.0 mV (Table S1).[2]

S2. Phantom MRI and optical imaging

MRI phantoms of the PSMA-targeted MNPs were prepared by mixing nanoparticles in 0.01 M PBS (100 µL) at different concentrations in 200 µL Eppendorf tubes. The PSMA-targeted MNP suspensions were then imaged with both T_2-weighted (T_2-W) MRI and optical imaging. Phantom MRI experiments were performed on a Bruker Biospec 11.7T horizontal bore scanner, equipped with a quadrature proton resonator radiofrequency coil. T_2-W images were acquired using a spin-echo pulse sequence: rapid acquisition with refocused echoes (RARE); echo time (TE) = 10 ms;

effective echo time (TE_{Eff}) = 30 ms; RARE factor = 8; repetition time (TR) = 2000 ms; number of averages (NA) = 2; field of view (FOV) = 25 × 25 mm; matrix size (MS) = 128 × 128 pixels; and slice thickness = 0.5 mm. Final image analyses were performed using the NIH ImageJ software. The changes in the T_2-W MRI signal from the MNPs in each phantom was calculated relative to that of a 0.01 M PBS phantom without MNPs. Phantom optical imaging experiments were performed using a LI-COR Pearl® Trilogy imaging system. Briefly, LI-COR IR Dye® 800CW on the PSMA-targeted MNPs was detected, using a fixed excitation wavelength of 785 nm and an emission wavelength of 800 nm. The images were acquired at a resolution of 170 μm. The fluorescence signal from each region of interest on the original image was then quantified using the LI-COR Pearl® Trilogy Imaging Software Version 2.0. The amount of optical signal enhancement from the MNPs in each optical phantom was calculated relative to that of a 0.01 M PBS phantom without MNPs.

Changes in the T_2-W MRI signal were detected in the phantoms, at MNP concentrations as low as 0.07 μg/μL (0.039 μg of Fe/μL), using T_2-W MRI (Figure S1). An exponential correlation was observed between the T_2-W MRI signal changes and the MNP concentration at high MNP concentrations. Optical signal changes were also detected in the phantoms, at MNP concentrations as low as 0.07 μg/μL (Figure S1). Similarly, an exponential correlation was observed between the optical signal change and the MNP concentration at high MNP concentrations. This similarity in the T_2-W MRI signal change trend and the optical image signal change trend suggested that the T_2-W MRI signal obtained from the PSMA-targeted MNP could be validated with its optical signal.

S3. Quantification of edema in PSMA(+) PC3 PIP tumors compared to PSMA(-) PC3 flu tumors using MRI bright pixel analysis.

Bright pixel analysis was performed, as previously reported.[3] Briefly, a 3D projection (brightest point) was generated from T_2-W MRIs of each mouse at each time-point, using the NIH ImageJ software. For each mouse, two regions of interest (ROIs) were next manually drawn over the PSMA(+) PC3 PIP and the PSMA(-) PC3 flu tumor regions, respectively, to cover the edematous areas (Figure S3A) at each time-point. Pixel intensity histograms were next generated for each ROI at each time-point. High intensity pixel thresholds that denoted the maximum difference between the two tumor phenotypes at each time-point after PDT were then manually chosen by comparing the pixel intensity histograms of the PSMA(+) PC3 PIP versus the PSMA(-) PC3 flu tumors at the different time-points (Figure S3B-D). The ratio of the number of bright pixels above the threshold in the PSMA(+) PC3 PIP versus the PSMA(-) PC3 flu tumor regions at each time-point was then computed to indicate the difference in edema between both tumor phenotypes.

Before PDT, at the 0 h time-point, there were no significant differences in the bright pixel signals between the PSMA(+) PC3 PIP and the PSMA(-) PC3 flu tumor regions. By taking the bright pixel signal ratio of the PSMA(+) PC3, PIP tumors to the PSMA(-) PC3 flu tumors in each mouse, a value of ~1 or ~100% was calculated for the 0 h time-point. Since bright pixels in the PSMA(+) PC3 PIP tumors increased compared to those in the PSMA(-) PC3 flu tumors at both the 18 h and the 42 h post-PDT time-points, the bright pixel signal ratio of the PSMA(+) PC3 PIP tumors to the PSMA(-) tumors also increased at both time-points.

Since at the 0 h time-point when a ratio of ~1 or ~100% was calculated, there was no difference in the bright pixel signals between PSMA(+) PC3 PIP and PSMA(-) PC3 flu tumors, a

ratio of 1 or 100% equals to zero signal difference. Thus, by subtracting the bright pixel signal ratio calculated for the 0 h time-point (~100%) from the ratios calculated for all the subsequent time-points post-PDT, a value of 0% was calculated for the 0 h time-point; X-100%; and Y-100% for the 18 h and 42 h post-PDT time-points respectively (Figure S3E). The statistics for the 18 h and the 42 h post-PDT time-points were computed by comparing the bright pixel signal ratio of the PSMA(+) PC3 PIP tumor region to the PSMA(-) PC3 flu tumor region at the respective time-points to that at the 0 h (pre-PDT) time-point.

S4. Quantification of edema in the PSMA(+) PC3 PIP tumor exterior versus the tumor interior by MRI bright pixel analysis.

To determine the ratio of edema in the PSMA(+) PC3 PIP tumor exterior versus that in the tumor interior, bright pixel analysis was performed. To facilitate the demarcation of the tumor interior from the tumor exterior, a 2D Z-projection (maximum) was generated from selected T_2-W MRI slices for each mouse at each time-point, using the NIH ImageJ software. Two regions of interest (ROIs) were next manually drawn over the tumor exterior and the tumor interior, respectively (Figure S4A). Pixel intensity histograms were then generated for each ROI at each time-point. A high intensity pixel threshold was then chosen by comparing the pixel intensity histograms of the tumor exterior and the tumor interior. A threshold value that denoted the maximum difference between the tumor exterior and the tumor interior at a given time-point post-PDT was then manually chosen (Figure S4B). The ratio of the number of signal intensity pixels above the threshold in the tumor exterior and the tumor interior, at each time-point, was then calculated as described above in Method S3 (Figure S4C).

S5. MRI black pixel analysis for dual tumor models

For Group 1 mice bearing both PSMA(+) PC3 PIP and PSMA(-) PC3 flu tumors, black pixel analysis was performed as previously reported.[2-5] Briefly, a 2D Z-projection of the minimum signal intensity was generated from selected T_2-weighted image slices, using the NIH ImageJ software. Two regions of interest (ROIs) were next manually drawn over the PSMA(+) and the PSMA(-) tumors, respectively (Figure S5A). Pixel intensity histograms were then generated for each ROI at each imaging time-point. Low intensity pixel thresholds were then chosen by comparing the pixel intensity histograms of the PSMA(+) tumors versus the PSMA(-) tumors at the different time-points. Threshold values that denoted the maximum difference between the two tumor phenotypes at a given time-point post-PDT were then manually chosen (Figure S5B-D). The ratio of the number of pixels below the threshold from both tumor phenotypes at each imaging time-point was then computed.

Prior to PDT at the 0 h time-point, the black pixel signal ratio of the PSMA(+) tumors to the PSMA(-) tumors in each mouse was normalized to a value of ~1 or ~100%. Since at the 0 h time-point when a ratio of ~1 or ~100% was calculated, there was no difference in black pixels between PSMA(+) PC3 PIP and PSMA(-) PC3 flu tumors, a ratio of 1 or 100% equals to zero difference. Thus, by subtracting the black pixel signal ratio calculated for the 0 h time-point (~100%) from the ratios calculated for all the subsequent time-points post-PDT, a value of 0% was calculated for the 0 h time-point; X-100%; and Y-100% for the 18 h and 42 h post-PDT time-points respectively (Figure S5E).

S6. Derivatization and validation of MRI black pixel analysis for single tumor models

A derivative of the MRI black pixel analyses described above for a dual tumor mouse model was adapted and validated for a single tumor mouse model and was used to evaluate mice bearing single tumors in Groups 2-5. Briefly, a 2D Z-projection of the minimum signal intensity was generated from selected T_2-weighted image slices, using the NIH ImageJ software. Two regions of interest (ROIs) were next manually drawn, one over the tumor and the other in the background region (Figure S11A). Pixel intensity histograms were then generated for each ROI at each imaging time-point. Low intensity pixel threshold values were next chosen by comparing the pixel intensity histograms of the tumor region versus that of the background region at the different time-points. Threshold values that denoted the maximum background intensity were next manually chosen for the different time-points (Figure S11B-D). The ratio of the number of tumor pixels below the maximum background pixel (threshold) at each imaging time-point was then computed.

Prior to PDT at the 0 h time-point, the black pixel ratio of the tumor in each mouse was normalized to an initial value (I) in percentages (I%). Since at the 0 h time-point when a ratio of I% was calculated, a ratio of I% equals to zero difference. Thus, by subtracting the black pixel ratio calculated for the 0 h time-point (I%) from the ratios calculated for all the subsequent time-points post-PDT, a value of 0% was calculated for the 0 h time-point; X% - I%; and Y% - I% for the 18 h and 42 h post-PDT time-points respectively (Figure S11E).

Supporting Figures

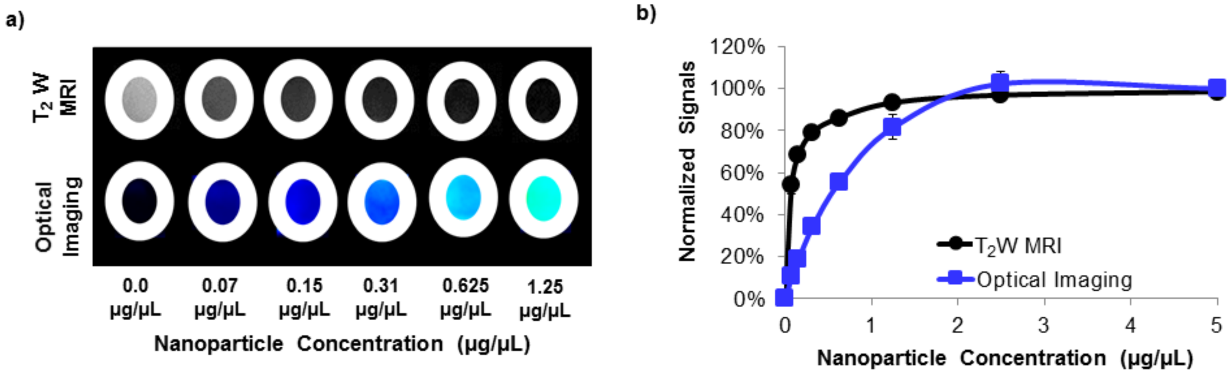

Figure S1. a) T_2-W MRIs (axial view) and optical images of phantoms containing PSMA-targeted MNPs at different nanoparticle concentrations. b) Graph of normalized T_2-W MRI and fluorescence signals generated by the PSMA-targeted MNPs present in the phantoms. The graph represents the normalized amount of signal changes in each phantom relative to that from a phantom without nanoparticles (blank phantom).

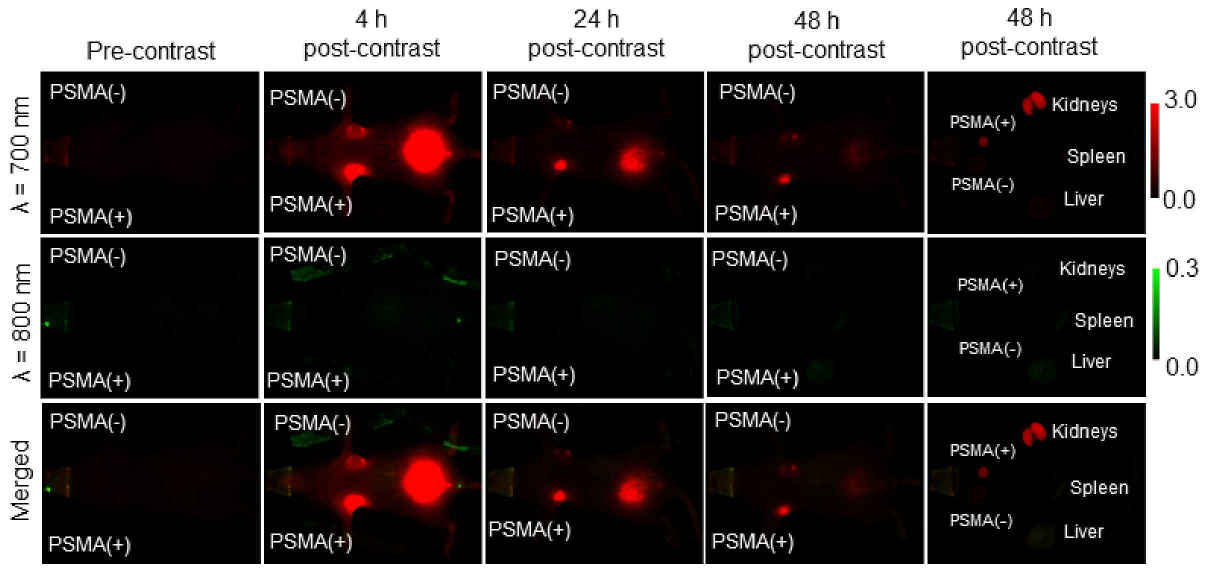

Figure S2. ***In vivo* fluorescence image-guided PDT.** 700 nm, 800 nm and merged *in vivo* fluorescence images of a representative male NSG mouse bearing both human PSMA(+) PC3 PIP

and PSMA(-) PC3 flu tumor xenografts, 0 h, 4 h, 24 h, and 48 h after intravenous administration of YC-9. Mice were irradiated with NIR light for PDT, 4 h after YC-9 administration.

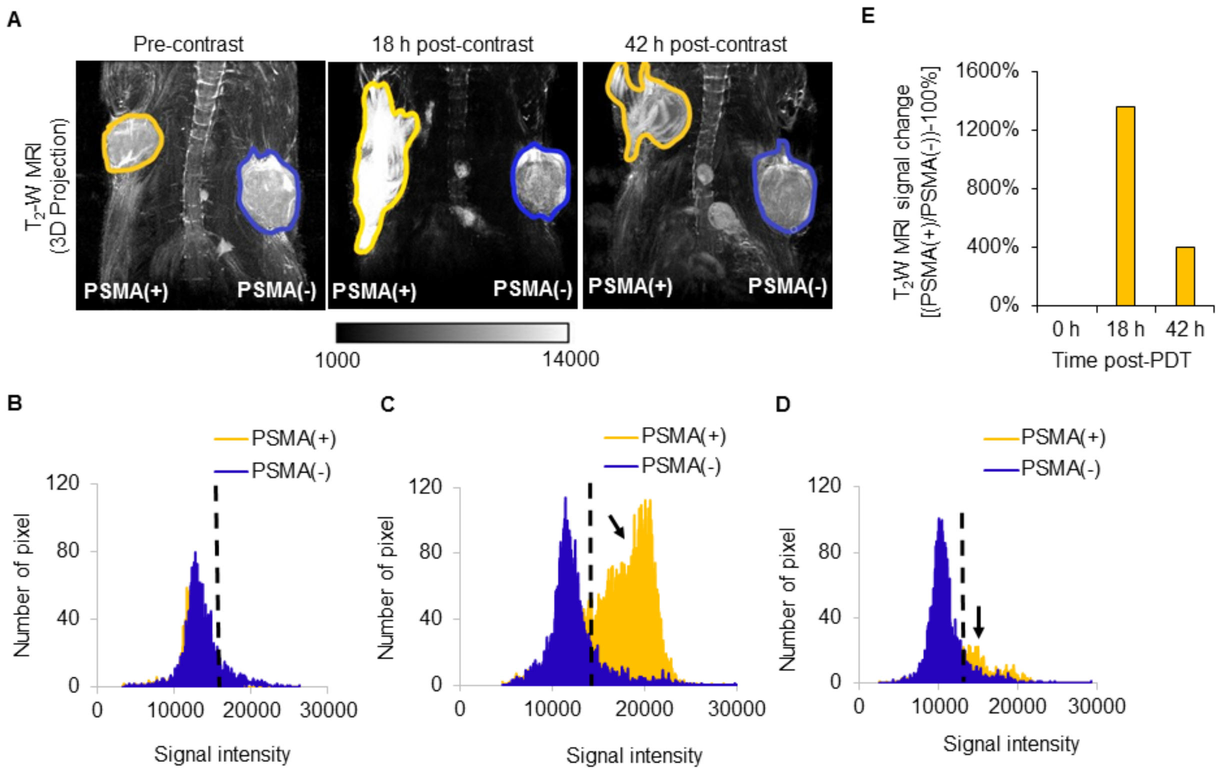

Figure S3. MRI bright pixel analysis of the tumor surroundings A) 3D projections (brightest point) from T_2-W MRIs, representative of a NSG mouse bearing human PSMA(+) PC3 PIP and PSMA(-) PC3 flu PC tumor xenografts; before PDT (0 h), 18 h and 42 h post-PDT. Twenty 0.5 mm slices were merged to form a single 3D projection image. Two regions of interest (ROIs) were then manually drawn over the PSMA(+) PC3 PIP and the PSMA(-) tumor surroundings, respectively. Signal intensity histograms generated from both PSMA(+) PC3 PIP and PSMA(-) PC3 flu tumors, **B)** before PDT (0 h), **C)** 18 h post-PDT and **D)** 42 h post-PDT. A high intensity pixel was chosen as a threshold bright pixel (dotted black line) for each time-point histogram. **E)** The ratio of the number of pixels above the bright intensity threshold in the PSMA(+) PC3 PIP

tumor compared to the PSMA(-)PC3 flu tumor 18 h and 42 h post-PDT was normalized against that before PDT (n=1).

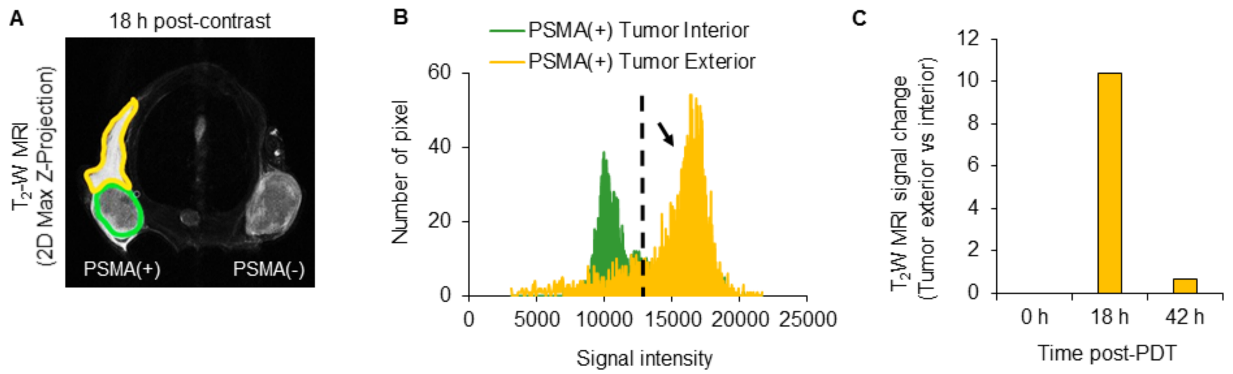

Figure S4. MRI bright pixel analysis of the tumor exterior versus interior A) 2D Z-projection (maximum) of a T_2-W MRI representative of a NSG mouse, bearing human PSMA(+) PC3 PIP and PSMA(-) PC3 flu tumor xenografts, 18 h post-PDT. Four 0.5 mm slices were merged to form a single 2D Z-projection image. Two regions of interest (ROIs) were then manually drawn over the tumor exterior and the tumor interior, respectively. **B)** A signal intensity histogram generated from the tumor exterior and the tumor interior 18 h post-PDT. A high intensity pixel was chosen as a threshold bright pixel (dotted black line) on the histogram. **C)** The ratio of the number of pixels above the bright intensity threshold in the tumor exterior and the tumor interior 18 h post-PDT, (n=1).

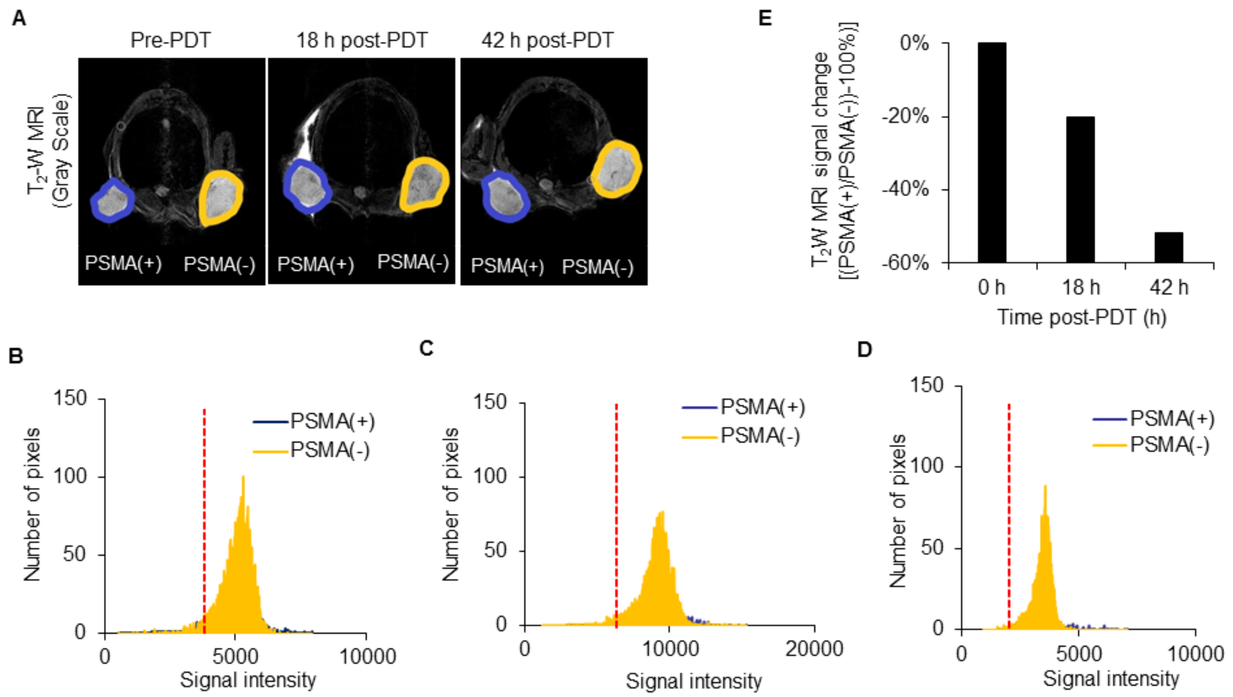

Figure S5. MRI black pixel analysis for a dual tumor mouse model A) A minimum 2D Z-projection of an axial T_2-W MRI, representative of a NSG mouse bearing human PSMA(+) PC3 PIP and PSMA(-) PC3 flu tumor xenografts: Pre-PDT, 18 h, and 42 h post-PDT. Four 0.5 mm slices were merged to form a single 2D Z-projection image. Two regions of interest (ROIs) were then manually drawn over the PSMA(+) PC3 PIP and PSMA(-) PC3 flu tumor xenografts, respectively. Signal intensity histogram generated from PSMA(+) PC3 PIP and PSMA(-) PC3 flu tumors **B)** Pre-PDT; **C)** 18 h post-PDT; **D)** 42 h post-PDT. A low intensity pixel was chosen as a threshold pixel (dotted red line) on the histogram. **E)** The ratio of the number of pixels below the pixel intensity threshold in the PSMA(+) PC3 PIP tumors compared to the PSMA(-) PC3 flu tumors was computed (n=1).

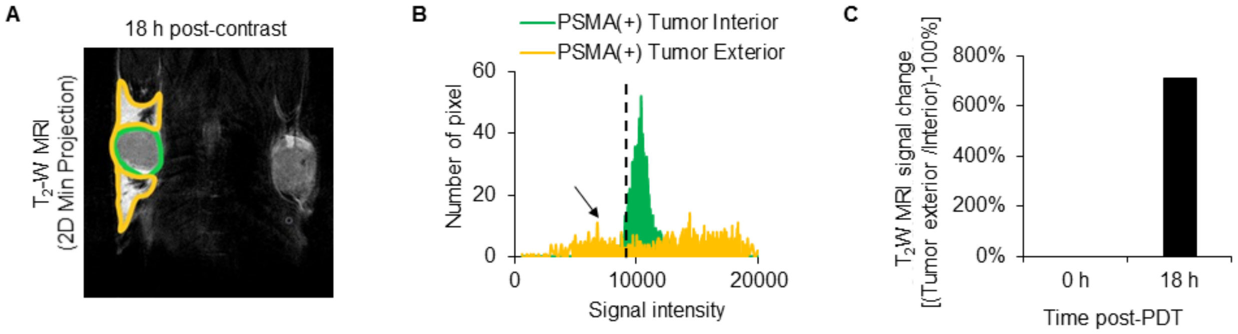

Figure S6. MRI black pixel analysis of the tumor exterior versus interior A) A minimum 2D Z-projection of a coronal T_2-W MRI representative of a NSG mouse, bearing human PSMA(+) PC3 PIP and PSMA(-) PC3 flu tumor xenografts, 18 h post-PDT. Four 0.5 mm slices were merged to form a single 2D Z-projection image. Two regions of interests (ROIs) were then manually drawn over the tumor exterior and the tumor interior, respectively. **B)** A signal intensity histogram generated from the tumor exterior and the tumor interior 18 h post-PDT. A low intensity pixel was chosen as a threshold pixel (dotted black line) on the histogram. **C)** The ratio of the number of pixels below the pixel intensity threshold in the tumor exterior and the tumor interior 18 h post-PDT, was computed (n=1).

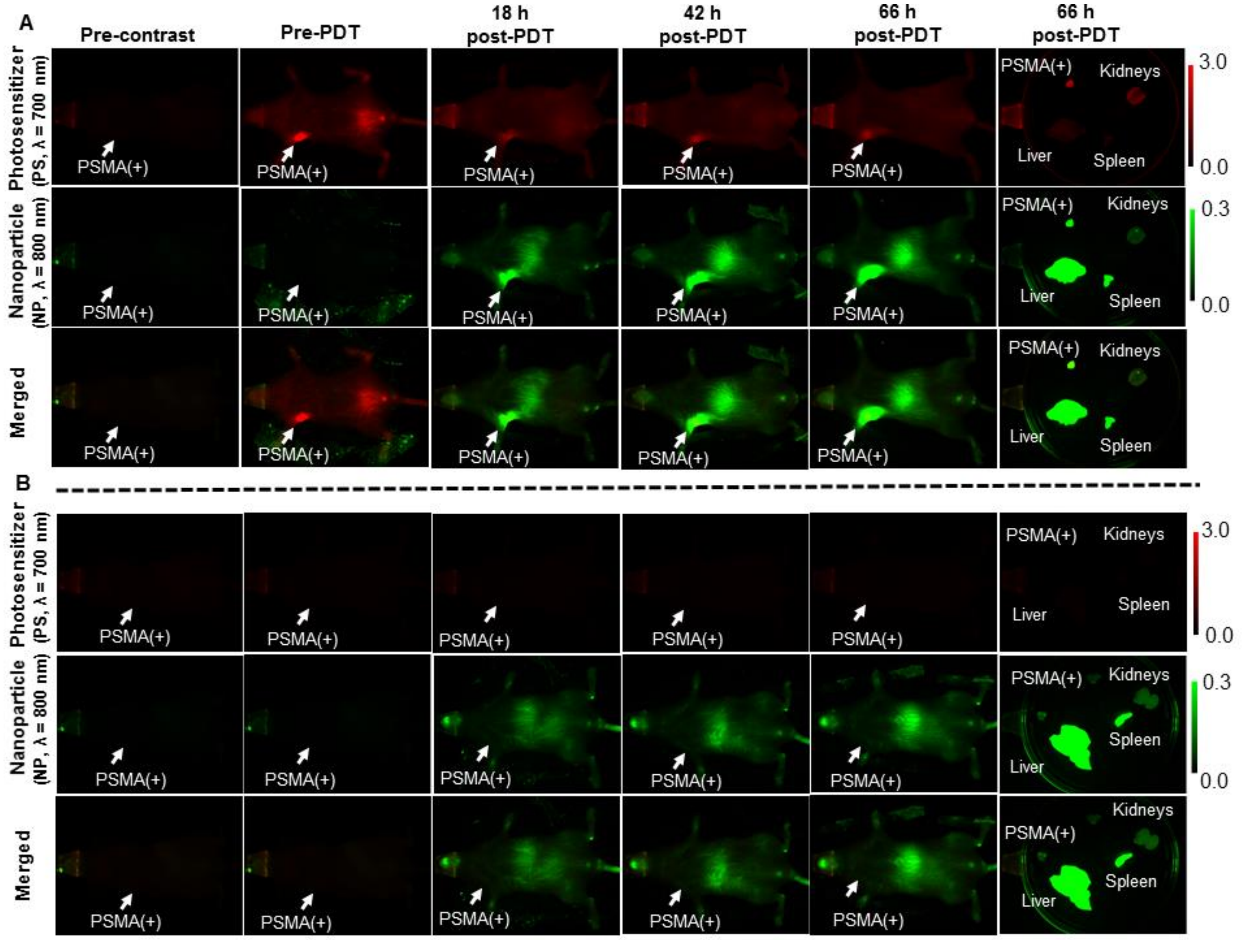

Figure S7. ***In vivo*** **fluorescence imaging of YC-9 (red) and MNP delivery (green) to Group 2 and Group 3 PSMA(+) PC3 PIP tumor-bearing mice. A)** *In vivo* fluorescence images (700 nm, 800 nm and merged) of a representative male NSG Group 2 mouse, bearing a PSMA(+) PC3 PIP tumor, pretreated with YC-9 (red) and PDT before PSMA-targeted MNP delivery (green). **B)** *In vivo* fluorescence images (700 nm, 800 nm and merged) of a representative male NSG Group 3 mouse, bearing a PSMA(+) PC3 PIP tumor, not treated with YC-9 (red) and PDT before PSMA-targeted MNP delivery (green).

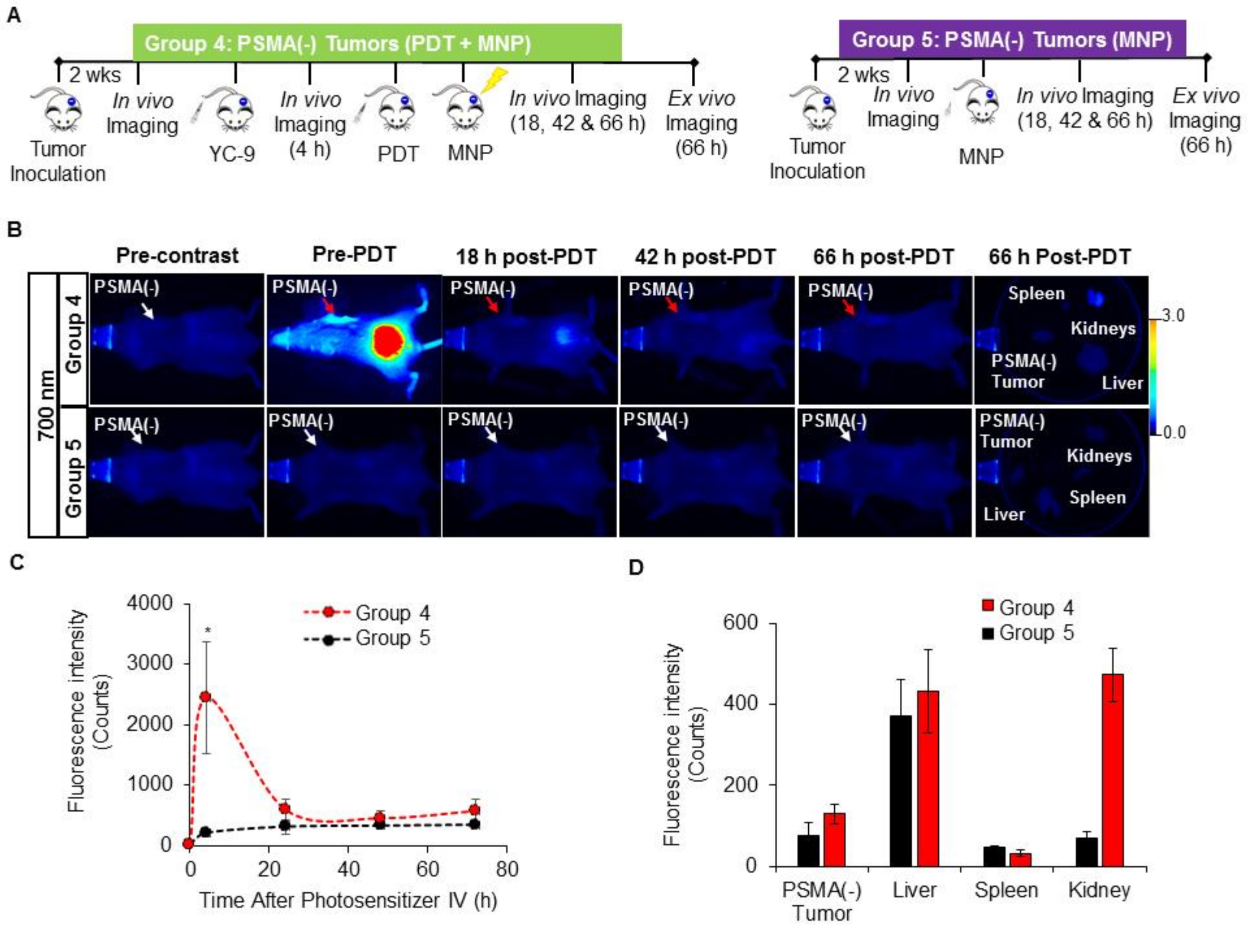

Figure S8. ***In vivo*** **fluorescence image-guided PDT in PSMA(-) PC3 flu tumor-bearing mice. A)** Schematic representing the experimental design for PSMA(-) PC3 flu tumor-bearing Groups 4 and 5 mice, respectively. Group 4 mice were administered 165 nmol/kg of YC-9, and the tumors were irradiated for PDT. Group 5 mice, on the other hand, were neither administered YC-9 nor irradiated for PDT. **B)** 700 nm *in vivo* fluorescence images of representative male NSG mice bearing human PSMA(-) PC3 flu tumor xenografts, from Group 4 and Group 5, respectively. **C)** Quantification of the 700 nm *in vivo* fluorescence signal in the PSMA(-) PC3 flu tumors of Group 4 mice compared to Group 5 mice (P = 0.017; n = 3), over 72 h post-YC-9 administration (66 h

post-PDT). **D)** Quantification of the 700 nm *ex vivo* fluorescence signal from the organs of Group 4 mice compared to those from Group 5 mice, 72 h after YC-9 administration (66 h post-PDT).

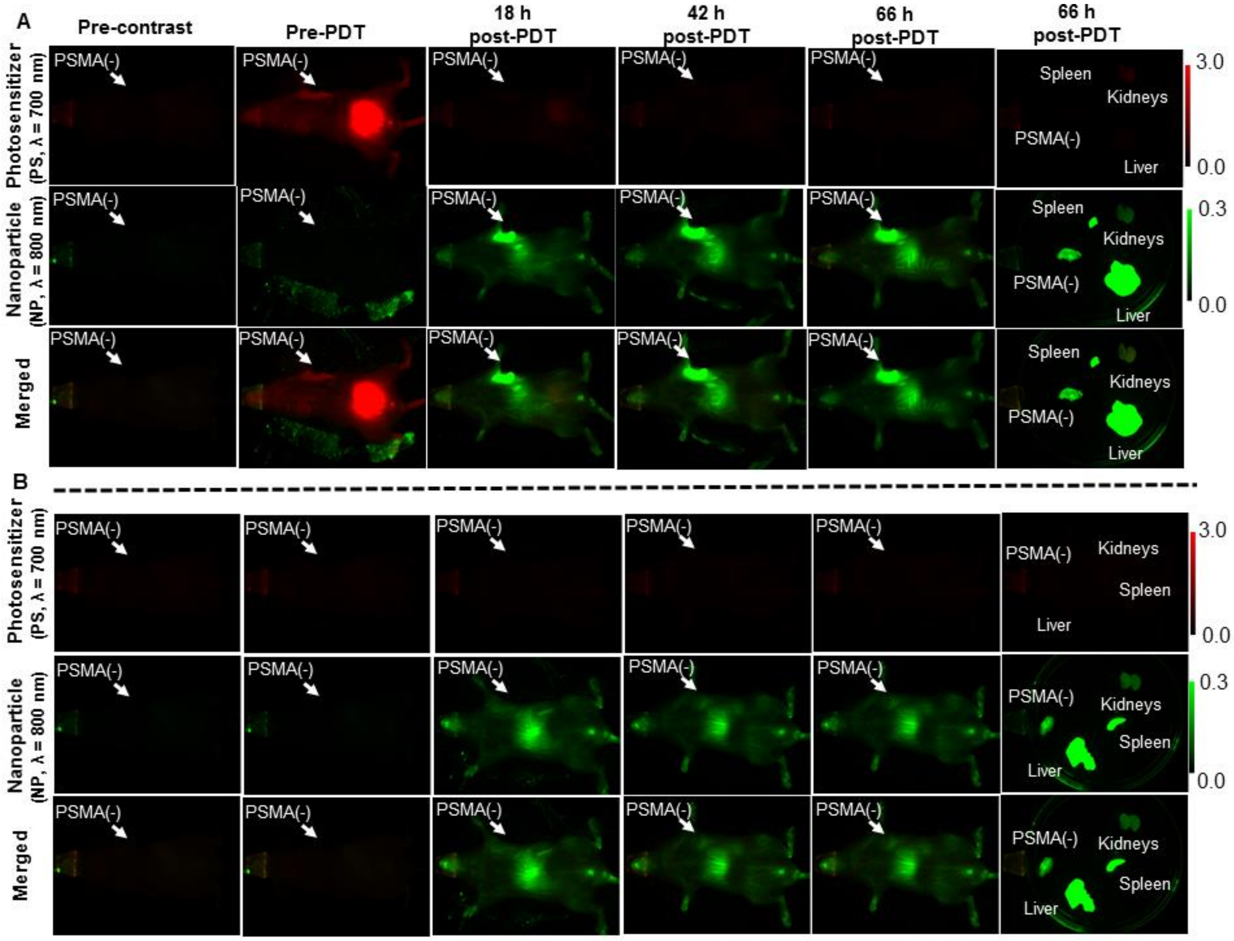

Figure S9. ***In vivo*** **fluorescence imaging of YC-9 (red) and MNP delivery (green) to Group 4 and Group 5 PSMA(-) PC3 flu tumor-bearing mice. A)** *In vivo* fluorescence images (700 nm, 800 nm and merged) of a representative male NSG Group 4 mouse, bearing a PSMA(-) PC3 flu tumor pretreated with YC-9 (red) and PDT before PSMA-targeted MNP delivery (green). **B)** *In vivo* fluorescence images (700 nm, 800 nm and merged) of a representative male NSG Group 5 mouse, bearing a PSMA(-) PC3 flu tumor, not pretreated with YC-9 (red) and PDT before PSMA-targeted MNP delivery (green).

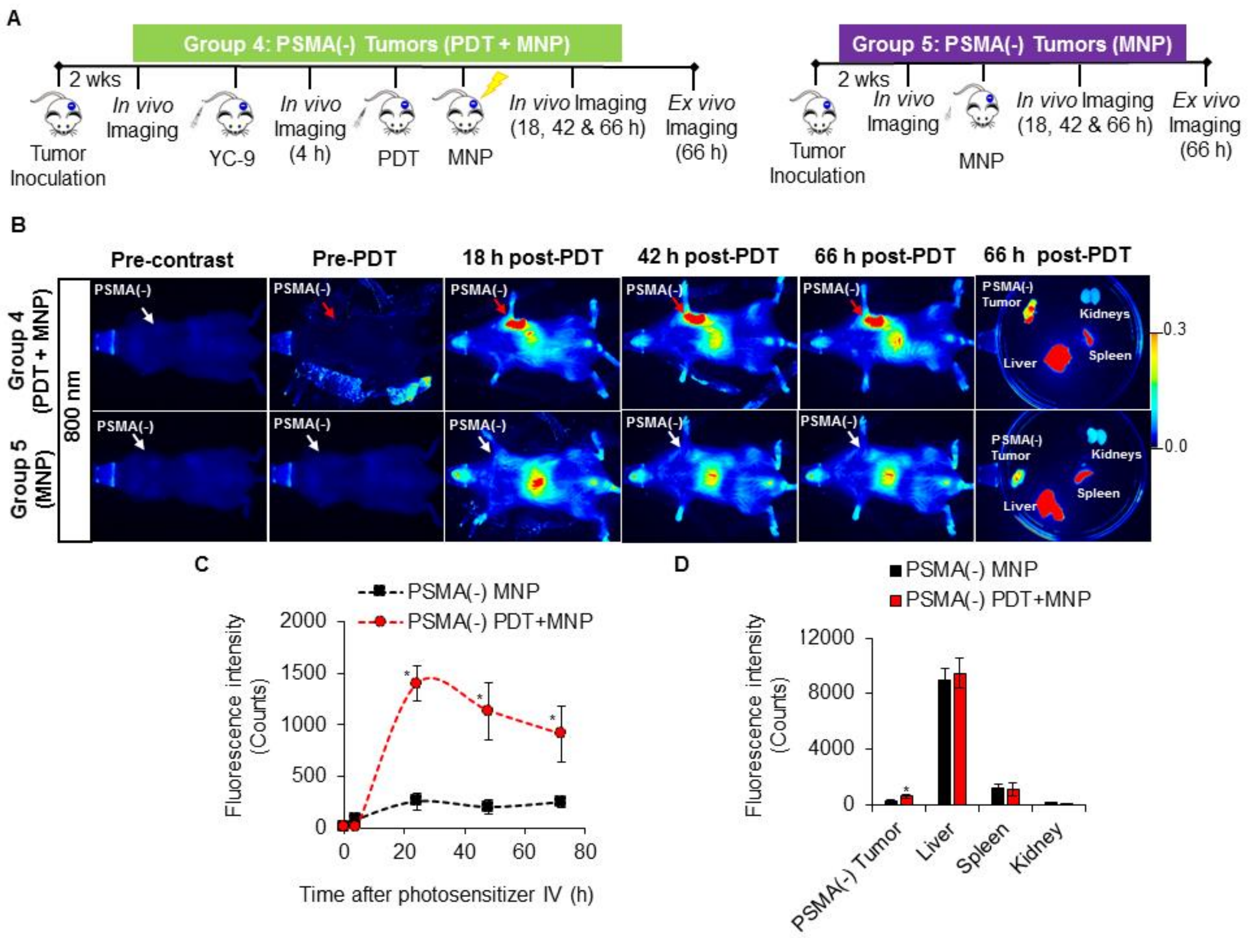

Figure S10. *In vivo* fluorescence imaging of enhanced MNP delivery to PSMA(-) PC3 flu tumors, after PDT. A) Schematic representing the experimental design for PSMA(-) PC3 flu tumor-bearing Groups 4 and 5 mice, respectively. **B)** 800 nm *in vivo* fluorescence images of representative male NSG mice bearing human PSMA(-) PC3 flu tumor xenografts, from Group 4 (PDT+MNP) and Group 5 (MNP), respectively. Group 4 mice were treated with PDT before the administration of the MNPs, while Group 5 mice were not treated with PDT. **C)** Quantification of the 800 nm *in vivo* fluorescence signal from the PSMA-targeted MNPs, in PSMA(-) PC3 flu tumors of Group 4 mice compared to those from Group 5 mice ($P \leq 0.018$; n = 3), over 66 h post-MNP administration. **D)** Quantification of the PSMA-targeted MNPs in the organs of Group 4

mice compared to those of Group 5 mice, using the 800 nm *ex vivo* fluorescence signal, 66 h after MNP administration (P = 0.020; n = 3).

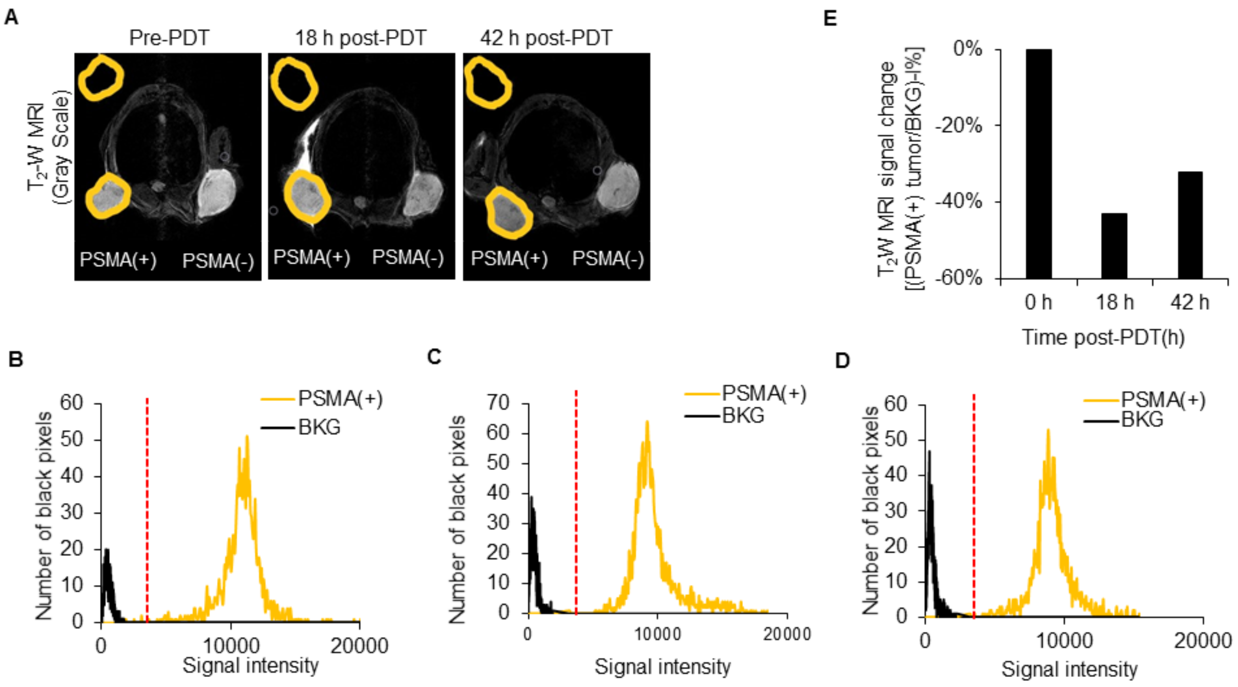

Figure S11. MRI black pixel analysis adaptation for a single tumor mouse model A) A minimum 2D Z-projection of an axial T_2-W MRI, representative of a NSG mouse bearing human PSMA(+) PC3 PIP and PSMA(-) PC3 flu tumor xenografts: Pre-PDT, 18 h post-PDT, and 42 h post-PDT. Four 0.5 mm slices were merged to form a single 2D Z-projection image. Two regions of interest (ROIs) were then manually drawn: One over the PSMA(+) PC3 PIP tumor and the other of the same size, over the background region. A signal intensity histogram was generated from both ROIs, **B)** Pre-PDT; **C)** 18 h post-PDT, and **D)** 42 h post-PDT. A threshold value that denoted the maximum background intensity was next manually chosen (dotted red line) on the histograms. **E)** The ratio of the number of pixels below the pixel intensity threshold in the PSMA(+) PC3 PIP tumor compared to the background was computed (n=1).

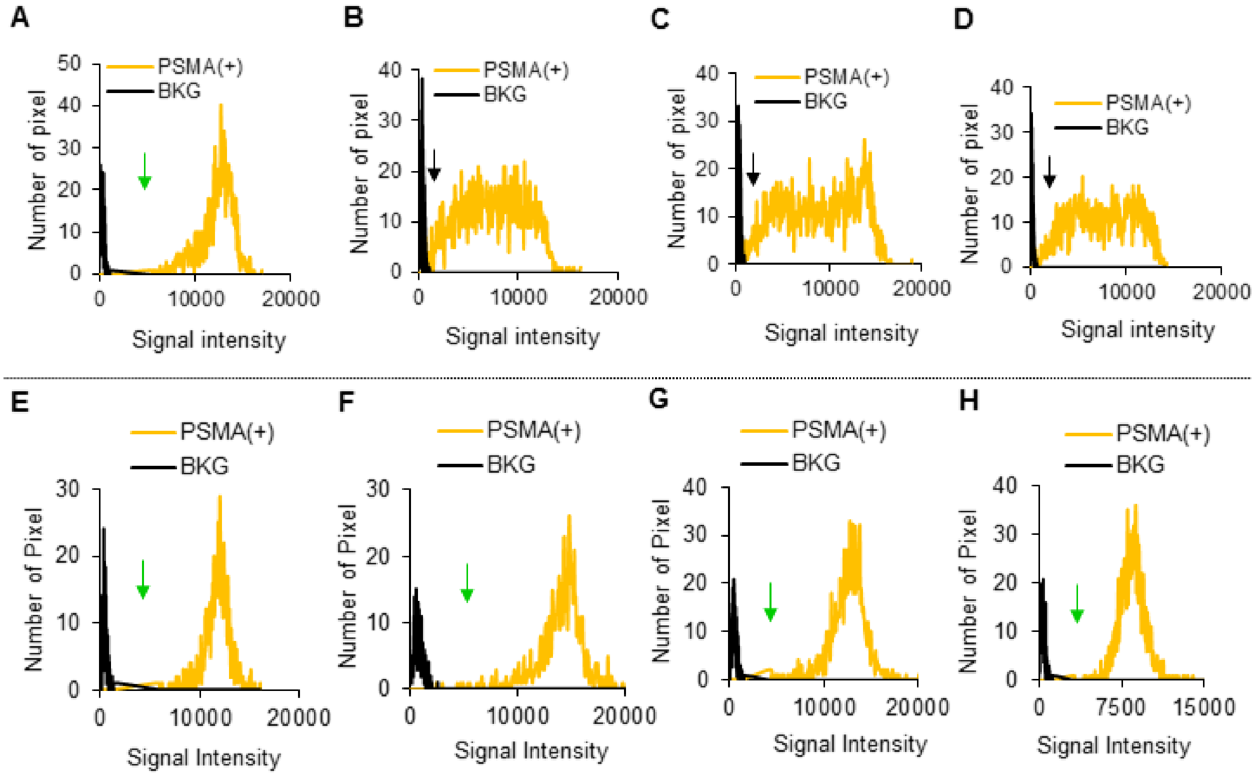

Figure S12. Histograms generated from *in vivo* MRI ROIs of a representative PSMA(+) PC3 PIP tumor and the background of representative Group 2 and Group 3 Mice. Pixel intensity histograms of PSMA(+) PC3 PIP tumor xenografts of a representative Group 2 mouse (pretreated with PDT), compared to the background (black) pixels: **A)** Pre-MNP; **B)** 18 h post-MNP; **C)** 42 h post-MNP, and **D)** 66 h post-MNP. Pixel intensity histograms of PSMA(+) PC3 PIP tumor xenografts of a representative Group 3 mouse (not treated with PDT), compared to the background (black) pixels: **E)** Pre-MNP; **F)** 18 h post-MNP; **G)** 42 h post-MNP; and **H)** 66 h post-MNP administration. The green arrows indicate a large difference between the tumor pixel distribution and the background (black) pixel distribution. The black arrows indicate a left shift in the tumor pixel intensity distribution towards background (black) pixels.

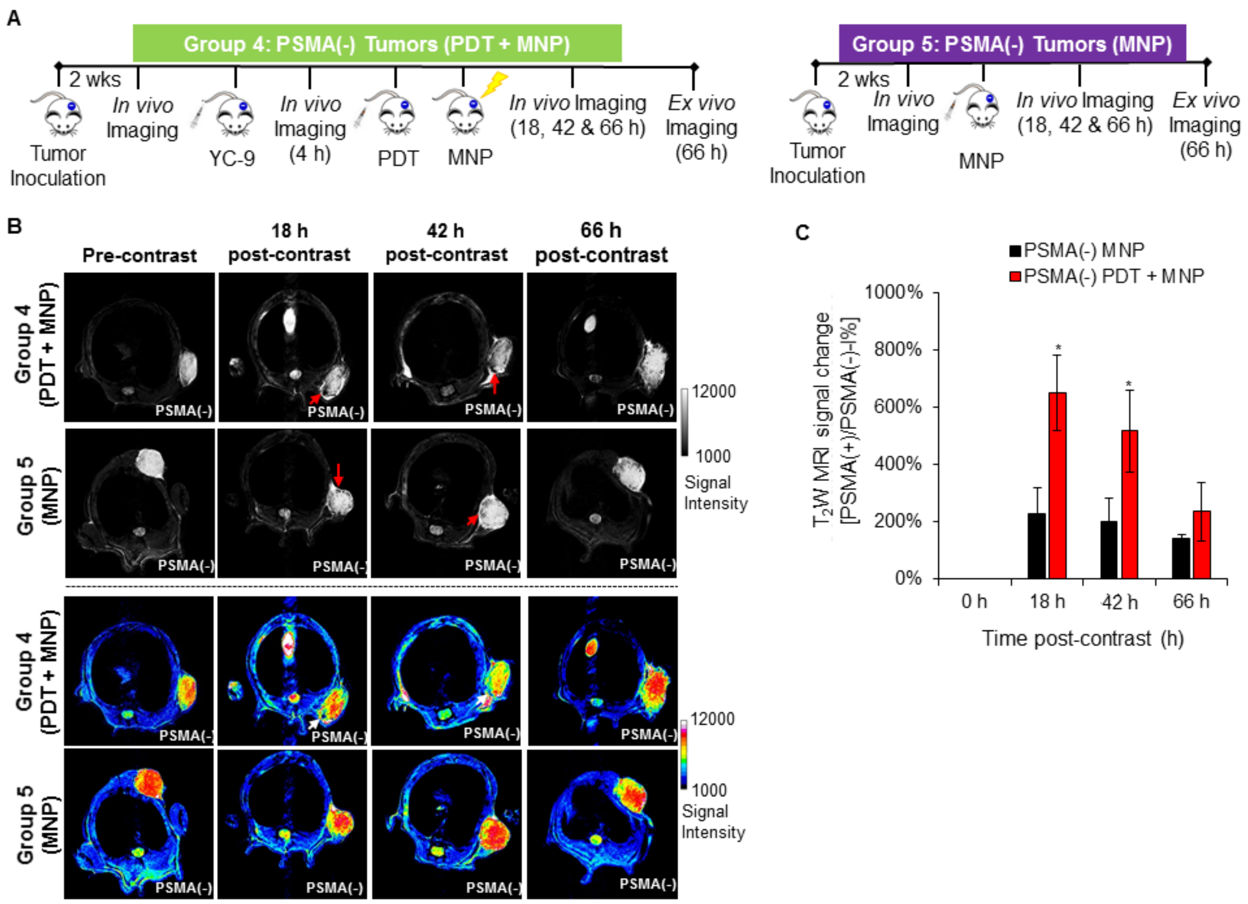

Figure S13. ***In vivo* MRI of MNP delivery to PSMA(-) PC3 flu tumors of representative Group 4 and Group 5 mice. A)** Schematic representing the experimental design for Groups 4 and 5 mice, respectively, and the T_2-W MRI schedule. **B)** *In vivo* MRI of representative male NSG mice bearing human PSMA(-) PC3 flu tumor xenografts, from Group 4 and Group 5, respectively, 0 h, 18 h, 42 h, and 66 h after the administration of PSMA-targeted MNPs. **C)** T_2-W MRI signal change ratios of PSMA(-) PC3 flu tumors in Group 4 mice compared to Group 5 mice, 0 h, 18 h, 42 h, and 66 h after MNP administration ($P \leq 0.041$; n = 3).

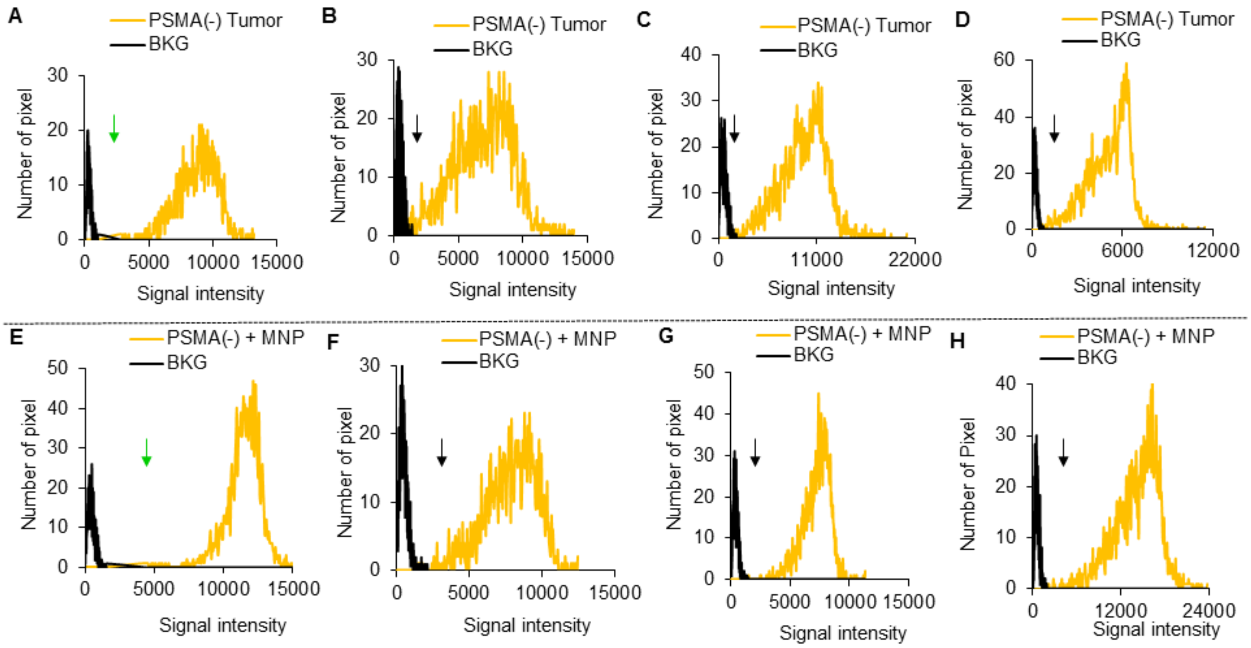

Figure S14. *In vivo* MRI of MNP delivery to PSMA(-) PC3 flu tumors of a representative Group 4 and Group 5 mouse. Pixel intensity histograms of PSMA(-) PC3 flu tumor xenografts of a representative Group 4 mouse (pretreated with PDT), compared to the background (black) pixels, **A)** before MNP; **B)** 18 h after MNP; **C)** 42 h after MNP; and **D)** 66 h after MNP administration. Pixel intensity histograms of PSMA(-) PC3 flu tumor xenografts of a representative Group 5 mouse (not treated with PDT), compared to the background (black) pixels, **E)** before MNP; **F)** 18 h after MNP; **G)** 42 h after MNP, and **H)** 66 h after MNP administration. The green arrows indicate a large difference between the tumor pixel distribution and the background (black) pixel distribution. The black arrows indicate a left shift in the tumor pixel intensity distribution towards background (black) pixels.

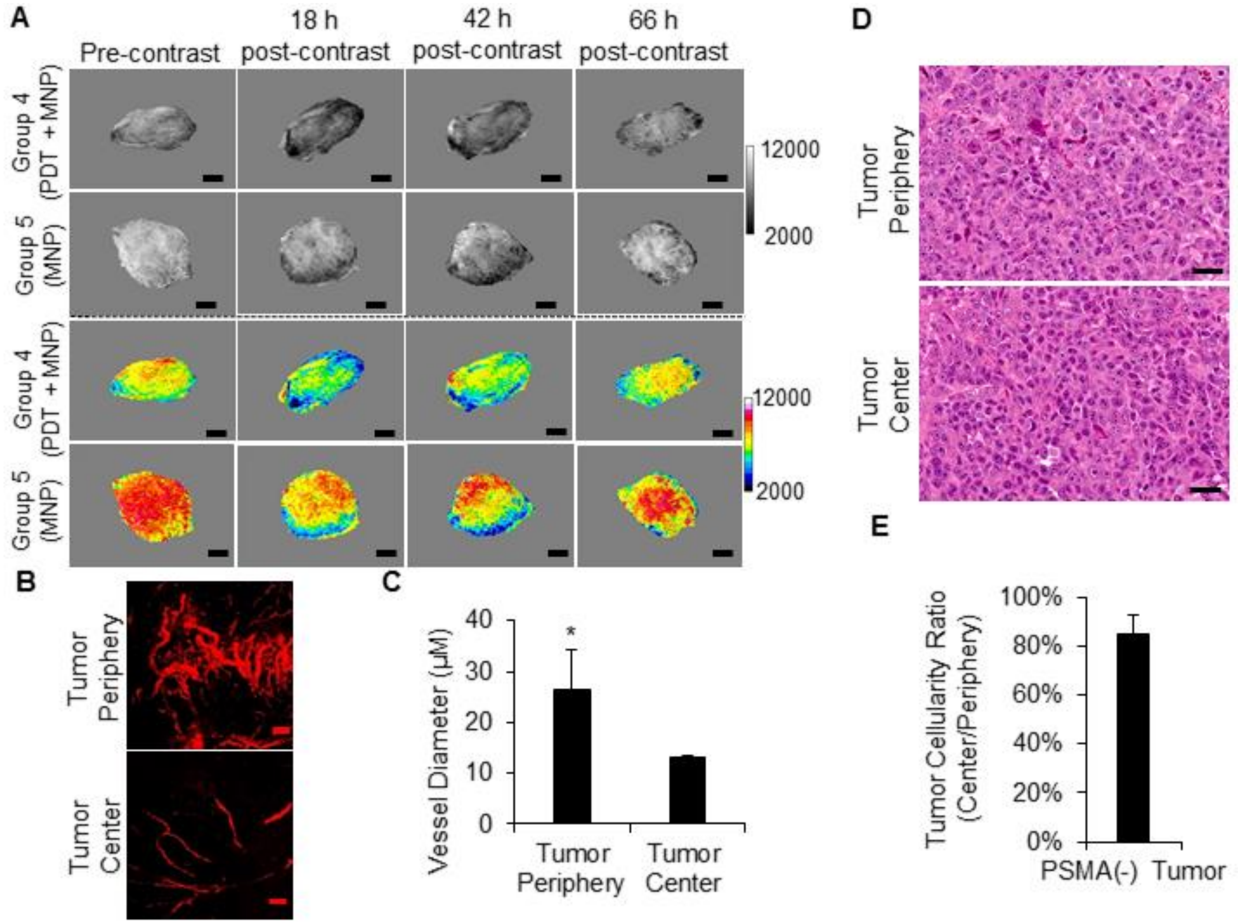

Figure S15. Imaging intra-tumoral MNP and vascular distribution: A) T_2-W MRIs (grayscale and colored) of the intra-tumoral signal change patterns of representative PSMA(-) PC3 flu tumors from Group 4 and Group 5, respectively. The signal change was generated from the delivered PSMA-targeted MNP in the tumor xenografts. **B)** Two-photon microscopy images of human PSMA(-) PC3 flu tumors, excised from mice after the intravenous administration of a 2,000 kDa Texas Red conjugated dextran polymer. The images show higher vascular densities at the tumor periphery compared to the tumor center. The scale bar represents 50 µm. **C)** Quantification of tumor blood vessel diameters at the tumor peripheries and the tumor centers. Blood vessels of larger diameters were found at the tumor periphery than the tumor center ($P = 0.026$; n = 3). **D)** Hematoxylin and eosin (H&E) staining of human PSMA(-) PC3 flu prostate tumors, excised from

untreated mice. The scale bar represents 50 µm. **E)** Quantification of the ratio of cellularity loss at the tumor center compared to the tumor periphery. This revealed no significant necrosis at either the tumor periphery or the tumor center.

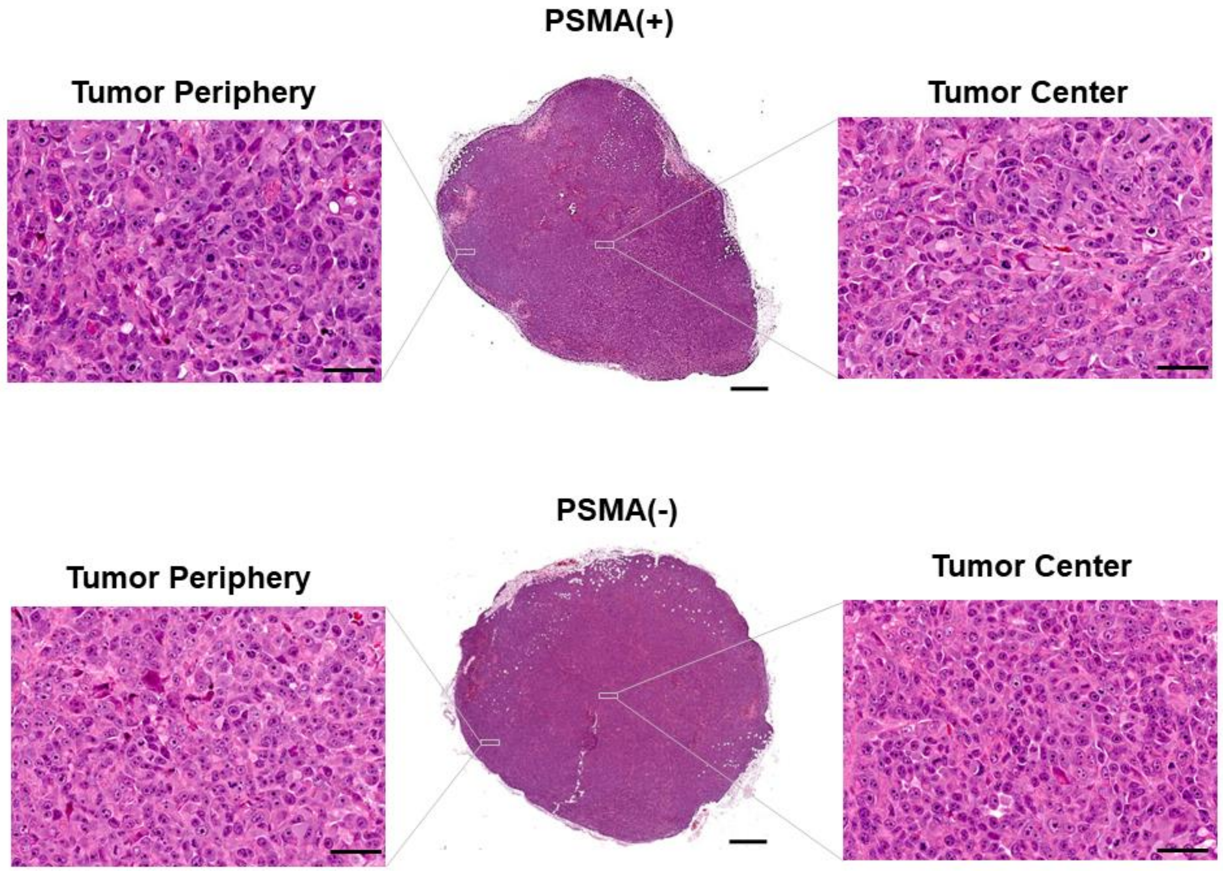

Figure S16. Hematoxylin and eosin (H&E) staining of human PSMA(+) PC3 PIP and PSMA(-) PC3 flu prostate tumors, at the approximate diameters used in this study. Both tumors were excised from untreated mice and exhibited comparable cellularity at both the tumor periphery and the tumor center. Center image scale (0.5 mm) and insert image scale (50 µm).

**Table S1.** Hydrodynamic diameters, polydispersity indices, and ζ-potentials of PSMA-targeted MNPs and unmodified MNPs measured using a Malvern ζ-sizer.			
	**Diameter (nm)**	**Polydispersity index**	**ζ-potential (mV)**
**PSMA-targeted MNP**	147 ± 8	0.08 ± 0.03	-10.9 ± 0.3
**Unmodified MNP**	133 ± 1	0.15 ± 0.07	+24.0 ± 2.0

**Table S2.** Organ biodistribution ratio from 800 nm *ex vivo* fluorescence images of Group 2 mice, 66 h post-PDT and PSMA-targeted MNP administration (Figure 6)			
**Organ**	**Mouse 1**	**Mouse 2**	**Mouse 3**
**PSMA(+)**	2.2%	3.4%	2.8%
**Liver**	84.4%	77.9%	77.1%
**Spleen**	12.0%	15.0%	16.3%
**Kidneys**	1.4%	3.7%	3.8%

**Table S3.** Organ biodistribution ratio from 800 nm *ex vivo* fluorescence images of Group 3 mice, 66 h after PSMA-targeted MNP administration (Figure 6)			
**Organ**	**Mouse 1**	**Mouse 2**	**Mouse 3**
**PSMA(+)**	0.3%	0.5%	1.0%
**Liver**	86.2%	87.3%	85.3%
**Spleen**	11.9%	9.8%	11.9%
**Kidneys**	1.6%	2.4%	1.8%

**Table S4.** Organ biodistribution ratio from 800 nm *ex vivo* fluorescence images of Group 4 mice, 66 h post-PDT and PSMA-targeted MNP administration (Figure S10)			
**Organ**	**Mouse 1**	**Mouse 2**	**Mouse 3**
**PSMA(-)**	6.1%	6.7%	4.2%
**Liver**	79.0%	80.2%	87.9%
**Spleen**	13.2%	8.1%	6.7%
**Kidneys**	1.7%	4.9%	1.1%

**Table S5.** Organ biodistribution ratio from 800 nm *ex vivo* fluorescence images of Group 5 mice, 66 h after PSMA-targeted MNP administration (Figure S10)			
**Organ**	**Mouse 1**	**Mouse 2**	**Mouse 3**
**PSMA(-)**	2.5%	3.2%	2.4%
**Liver**	85.6%	79.8%	85.3%
**Spleen**	9.7%	14.8%	10.3%
**Kidneys**	2.2%	2.1%	2.1%

References

1. Behnam Azad B, Banerjee SR, Pullambhatla M, Lacerda S, Foss CA, Wang Y, Ivkov R, Pomper MG: **Evaluation of a PSMA-targeted BNF nanoparticle construct**. *Nanoscale* 2015, **7**(10):4432-4442.
2. Ngen EJ, Benham Azad B, Boinapally S, Lisok A, Brummet M, Jacob D, Pomper MG, Banerjee SR: **MRI assessment of prostate-specific membrane antigen (PSMA) targeting by a PSMA-targeted magnetic nanoparticle: Potential for image-guided therapy**. *Mol Pharm* 2019, **16**(5):2060-2068.
3. Ngen EJ, Wang L, Kato Y, Krishnamachary B, Zhu W, Gandhi N, Smith B, Armour M, Wong J, Gabrielson K *et al*: **Imaging transplanted stem cells in real time using an MRI dual-contrast method**. *Sci Rep* 2015, **5**:13628.
4. Ngen EJ, Wang L, Gandhi N, Kato Y, Armour M, Zhu W, Wong J, Gabrielson KL, Artemov D: **A preclinical murine model for the early detection of radiation-induced brain injury using magnetic resonance imaging and behavioral tests for learning and memory: with applications for the evaluation of possible stem cell imaging agents and therapies**. *J Neurooncol* 2016, **128**(2):225-233.
5. Ngen EJ, Kato Y, Artemov D: **Direct cell labeling to image transplanted stem cells in real time using a dual-contrast MRI technique**. *Curr Protoc Stem Cell Biol* 2017, **42**(1):5A.10.11-15A.10.19.
